# Targeting signaling and apoptotic pathways involved in chemotherapeutic drug-resistance of hematopoietic cells

**DOI:** 10.18632/oncotarget.20408

**Published:** 2017-08-24

**Authors:** Stephen L. Abrams, Peter P. Ruvolo, Vivian R. Ruvolo, Giovanni Ligresti, Alberto M. Martelli, Lucio Cocco, Stefano Ratti, Agostino Tafuri, Linda S. Steelman, Saverio Candido, Massimo Libra, James A. McCubrey

**Affiliations:** ^1^ Department of Microbiology and Immunology, Brody School of Medicine, East Carolina University, Greenville, NC, USA; ^2^ Section of Signal Transduction and Apoptosis, Hormel Institute, University of Minnesota, Austin, MN, USA; ^3^ Current/Present address: Department of Leukemia, The University of Texas M. D. Anderson Cancer Center, Houston, TX, USA; ^4^ Department of Biomedical and Biotechnological Sciences, Pathology and Oncology Section, University of Catania, Catania, Italy; ^5^ Current/Present address: Department of Physiology and Biomedical Engineering, Mayo Clinic College of Medicine, Rochester, MN, USA; ^6^ Department of Biomedical and Neuromotor Sciences, Università di Bologna, Bologna, Italy; ^7^ Hematology, Department of Clinical and Molecular Medicine, Sant’Andrea Hospital, Sapienza University of Rome, Rome, Italy

**Keywords:** TP53, chemosensitivity, drug sensitivity, apoptosis, nutlin-3a

## Abstract

A critical problem in leukemia as well as other cancer therapies is the development of chemotherapeutic drug-resistance. We have developed models of hematopoietic drug resistance that are based on expression of dominant-negative TP53 [TP53 (DN)] or constitutively-active MEK1 [MEK1(CA)] oncogenes in the presence of chemotherapeutic drugs. In human cancer, functional TP53 activity is often lost in human cancers. Also, activation of the Raf/MEK/ERK pathway frequently occurs due to mutations/amplification of upstream components of this and other interacting pathways. FL5.12 is an interleukin-3 (IL−3) dependent hematopoietic cell line that is sensitive to doxorubicin (a.k.a Adriamycin). FL/Doxo is a derivative cell line that was isolated by culturing the parental FL5.12 cells in doxorubicin for prolonged periods of time. FL/Doxo + TP53 (DN) and FL/Doxo + MEK1 (CA) are FL/Doxo derivate cell lines that were infected with retrovirus encoding TP53 (DN) or MEK1 (CA) and are more resistant to doxorubicin than FL/Doxo cells. This panel of cell lines displayed differences in the sensitivity to inhibitors that suppress mTORC1, BCL2/BCLXL, MEK1 or MDM2 activities, as well as, the proteasomal inhibitor MG132. The expression of key genes involved in cell growth and drug-resistance (e.g., MDM2, MDR1, BAX) also varied in these cells. Thus, we can begin to understand some of the key genes that are involved in the resistance of hematopoietic cells to chemotherapeutic drugs and targeted therapeutics.

## INTRODUCTION

We have been investigating the roles of signal transduction pathways in chemotherapeutic drug- and radiation-resistance as well as sensitivity to small molecule inhibitors. The RAF/MEK/ERK and PI3K/PTEN/AKT/mTORC1 pathways are central signal transduction pathways that are often activated in human cancers due to various mechanisms, including dysregulation of intrinsic components, as well as, mutations in upstream receptors and other interacting signaling molecules. These pathways are also regulated by other mechanisms including epigenetic mechanisms, post-translation modification and micro-RNAs (miRs) which alter the expression of key components as well as downstream effector molecules [1]. We have determined that some signaling pathways such as RAF/MEK/ERK, PI3K/PTEN/AKT/mTORC1 and TP53 are critically involved in the sensitivity of various cancers such as; hematopoietic, breast, prostate and pancreatic to chemotherapy, radiotherapy and targeted therapy [2–8].

We determined that overexpression of constitutively-active or conditionally-active signaling molecules, (*e.g*., HA-RAS, RAF, MEK1, PI3K, AKT, GSK-3beta, ABL and BCR-ABL) and upstream growth factor receptors (*e.g*., epidermal growth factor receptor [EGFR] or insulin like growth factor receptor [IGF-1R] will alter the cytokine-dependency of hematopoietic or doxorubicin-sensitivity of breast or prostate cancer cells [7–12]. Introduction of phosphatase-deficient forms of PTEN or kinase-dead GSK-3beta will also alter the sensitivity of breast cancer cells to chemotherapeutic drugs and in some cases hormonal based therapies such as tamoxifen and targeted therapies such as rapamycin [5, 6, 10]. The RAF/MEK/ERK pathway will also induce the expression of cytokines and cytokine receptors which can alter the growth properties of cancer cells [8] and the drug- resistance of breast cancer cells [8–12]. The RAF/MEK/ERK, PI3K/PTEN/AKT/mTORC1, EGFR, IGF-1R and other signaling pathways can also induce key proteins involved in regulation of cell cycle progression and resistance to chemotherapeutic drugs [13–25]. The RAF and PI3K pathways have also been linked with epithelial to mesenchymal (EMT) transition in breast cancer cell lines [10, 11].

TP53 is a critical transcription factor which is regulated by multiple mechanisms [19–21]. TP53 is referred to as a tumor suppressor gene and it is frequently mutated and inactivated in human cancer. TP53 is also regulated by multiple mechanisms, including transcriptional, post-translational and microRNAs (miRs). TP53 also regulates the expression of multiple miRs, especially miR-34 which is a tumor suppressor miR. Previously we determined that TP53 was important in the drug-resistance of hematopoietic cells and involved in controlling the sensitivity of prostate cancer cells to chemotherapy as well as radiation therapy [22, 23].

Anti-apoptotic proteins such as BCL2, BCLXL, MCL1, X-linked inhibitor of apoptosis (XIAP1) are also involved in drug-resistance [24–26]. Inhibitors to certain anti-apoptotic proteins have been developed [26]. Drug-resistance proteins serve multiple roles, including efflux of xenobiotics as well as chemotherapeutic drugs. These proteins are also regulated by signaling cascades such as PI3K/PTEN/AKT/mTORC1 and RAF/MEK/ERK pathways [27–33]. There are also interactions between the RAF/MEK/ERK pathways and BCL2 proteins which can influence growth properties of hematopoietic and breast cancer cells [31–33]. Targeting signal transduction and apoptotic molecules is a therapeutic approach used in many cancers and cancer stem cells as well as in the suppression of aging [34–69].

The FL5.12 cell line represents a well-established interleukin-3 (IL-3) hematopoietic model which has been used to study key genes involved in hematopoietic and leukemia growth [70]. Previously we determined that the FL5.12 cell line was sensitive to doxorubicin [21]. Doxorubicin-resistant FL/Doxo cells were isolated from the parental FL5.12 cells by sub culturing the FL5.12 cells in 10 nM doxorubicin for three months [21]. FL/Doxo cells were also infected with retroviruses encoding dominant negative (DN) TP53 [TP53 (DN)] or constitutively-active MEK1 [MEK1 (CA)] [21]. These two modified FL/Doxo cell lines were more resistant to doxorubicin than the FL/Doxo cells from which they were derived. This panel of cells provides useful models to understand some of the effects of mutated genes on the sensitivity to chemotherapeutic drugs and signal transduction inhibitors. In this manuscript, we have compared the sensitivities of these cells to a series of signal transduction inhibitors which target the proteasome, the RAF/MEK/ERK, PI3K/PTEN/AKT/mTORC1, TP53 (MDM2) pathways and BCL2/BCLXL anti-apoptotic proteins. Moreover, the effects of various targeted therapeutics were examined when they were combined with the chemotherapeutic drug doxorubicin or other small molecule inhibitors.

## RESULTS

### Differential sensitivity to the proteasomal inhibitor MG132

**Figure 1:**
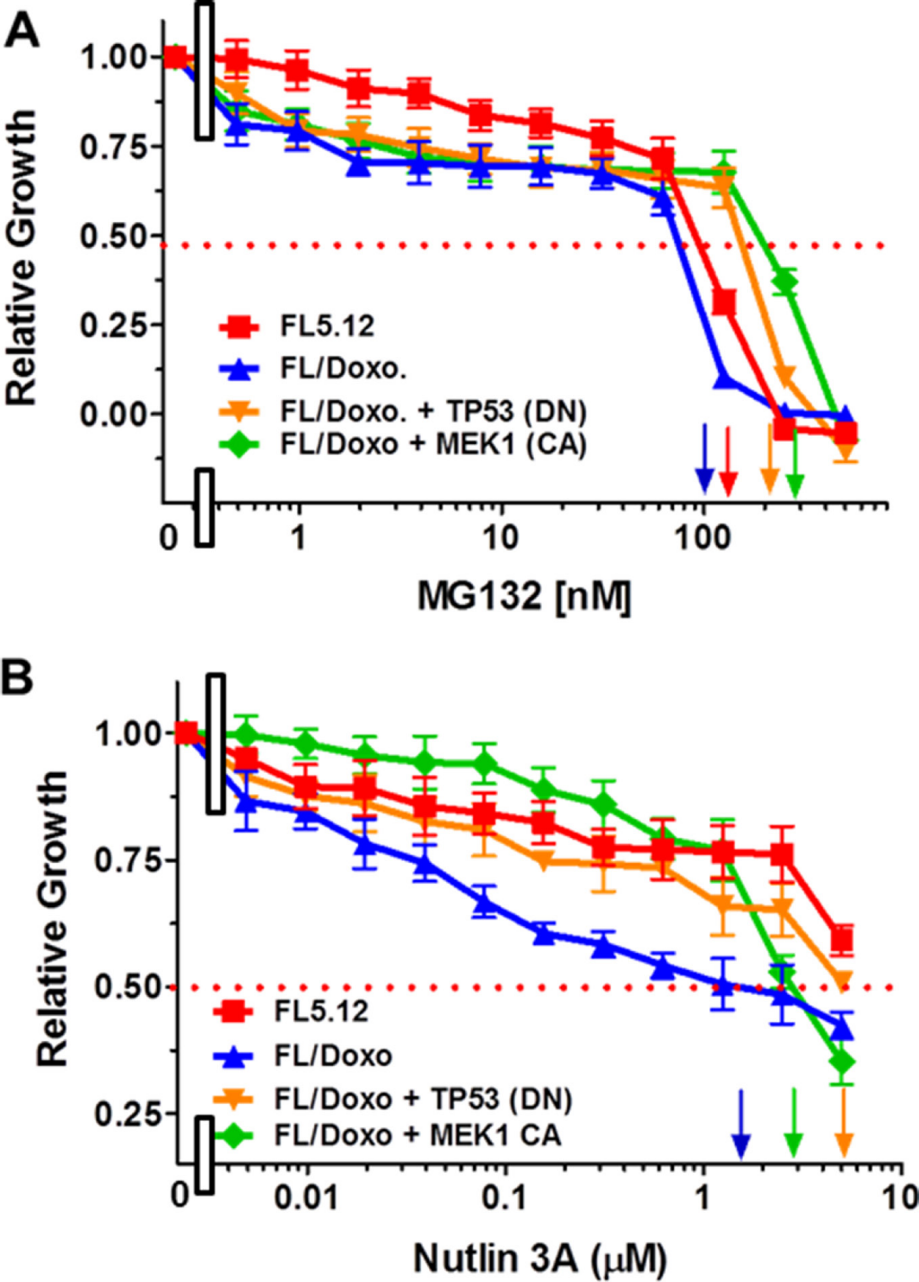
Effects of the Proteasomal Inhibitor MG132 and MDM2 Inhibitor Nutlin-3a on FL5.12, FL/Doxo, FL/Doxo + TP53 (DN) and FL/Doxo + MEK1 (CA) Cells The effects of these inhibitors on: FL5.12 (solid red squares), FL/Doxo (solid blue upward triangles), FL/Doxo + TP53 (DN) (solid orange downward diamonds) and FL/Doxo + MEK1 (CA) (solid green diamonds) cells were determined by titrating all of the cells on the same day with: MG132 (Panel **A**) and Nutlin-3A (Panel **B**) inhibitors and then examined four days later by MTT analysis as described [21]. Arrows pointing to the X-axis indicate where the IC_50_ can be estimated. Statistical analysis (unpaired *t* test results) indicated that the two-tailed *P* values for FL/Doxo + MEK1(CA) and FL/Doxo + TP53 (DN) vs FL/Doxo in Panel A were less than 0.0001 which is considered to be extremely statistically significant. The two-tailed *P* value for FL5.12 vs FL/Doxo in Panel A equaled 0.0026 which is considered to be very statistically significant. In Panel B, the *P* value between the FL/Doxo + TP53 (DN) and FL/Doxo was determined to be less than 0.0001 which is considered to be extremely highly significant. These experiments were performed four times with similar results.

The sensitivities of the FL5.12, FL/Doxo, FL/Doxo + TP53 (DN) and FL/Doxo + MEK1 (CA) cells to the proteasomal inhibitor MG132 were determined. All the four different cell lines were examined the same day. The FL/Doxo + TP53 (DN) and FL/Doxo + MEK1 (CA) cells were more resistant to the proteasomal inhibitor MG-132 than the FL5.12 and FL/Doxo cells as IC_50_s of approximately 200 nM, 300 nM, 120 nM and 100 nM were observed, respectively. Introduction of the *TP53* (DN) gene increased the resistance of the FL/Doxo + TP53 (DN) cells approximately 1.7- to 2-fold compared to the FL5.12 and FL/Doxo cells respectively (Figure 1A). Introduction of the constitutively-active *MEK1* (CA) gene increased the resistance of the FL/Doxo + MEK1 (CA) cells approximately 2.5- to 3-fold respectively compared to the FL5.12 and FL/Doxo cells (Figure 1A). Suppression of the proteasome by the proteasomal inhibitor results in the stabilization of TP53 WT [21]. Other studies have observed that proteasomal inhibition leads to increased TP53 nuclear levels and also results in induction of G_1_ arrest, apoptosis, and TP53-mediated gene expression (*e.g*., p21^Cip1^, PUMA, BAX) [71]. Cells which expressed TP53 (DN) or MEK1 (CA) were more resistant to the proteasomal inhibitor (Figure 1A) suggesting that the presence of the TP53 (DN) and MEK1 (CA) could abrogate some of the effects of the proteasomal inhibitor. Combining MG-132 and other proteasomal inhibitors with signal transduction inhibitors has been shown to be effective in inducing death in certain cancers [71–73].

### Differential sensitivity to MDM2 inhibitor, nutlin-3a

Nutlin-3a is a small molecule inhibitor that targets MDM2 [74, 75]. FL/Doxo cells were more sensitive to treatment with the nutlin-3a (IC_50_ = 1.5 μM) than either FL5.12 or FL/Doxo + TP53 (DN) cells (Figure 1B). Approximately 5 μM nutlin-3a was required to reach the IC_50_ of the FL5.12 and FL/Doxo + TP53 (DN) cells. The FL/Doxo + MEK1 (CA) cells were more sensitivity to nutlin-3a as an IC_50_ of approximately 3 μM was observed. FL/Doxo and FL/Doxo + MEK1 (CA) cells express functional TP53 [21]. Thus, the FL/Doxo cells were more sensitivity to agents which could alter TP53 or MDM2 activity.

### Differential sensitivity to mapk inhibitors

**Figure 2:**
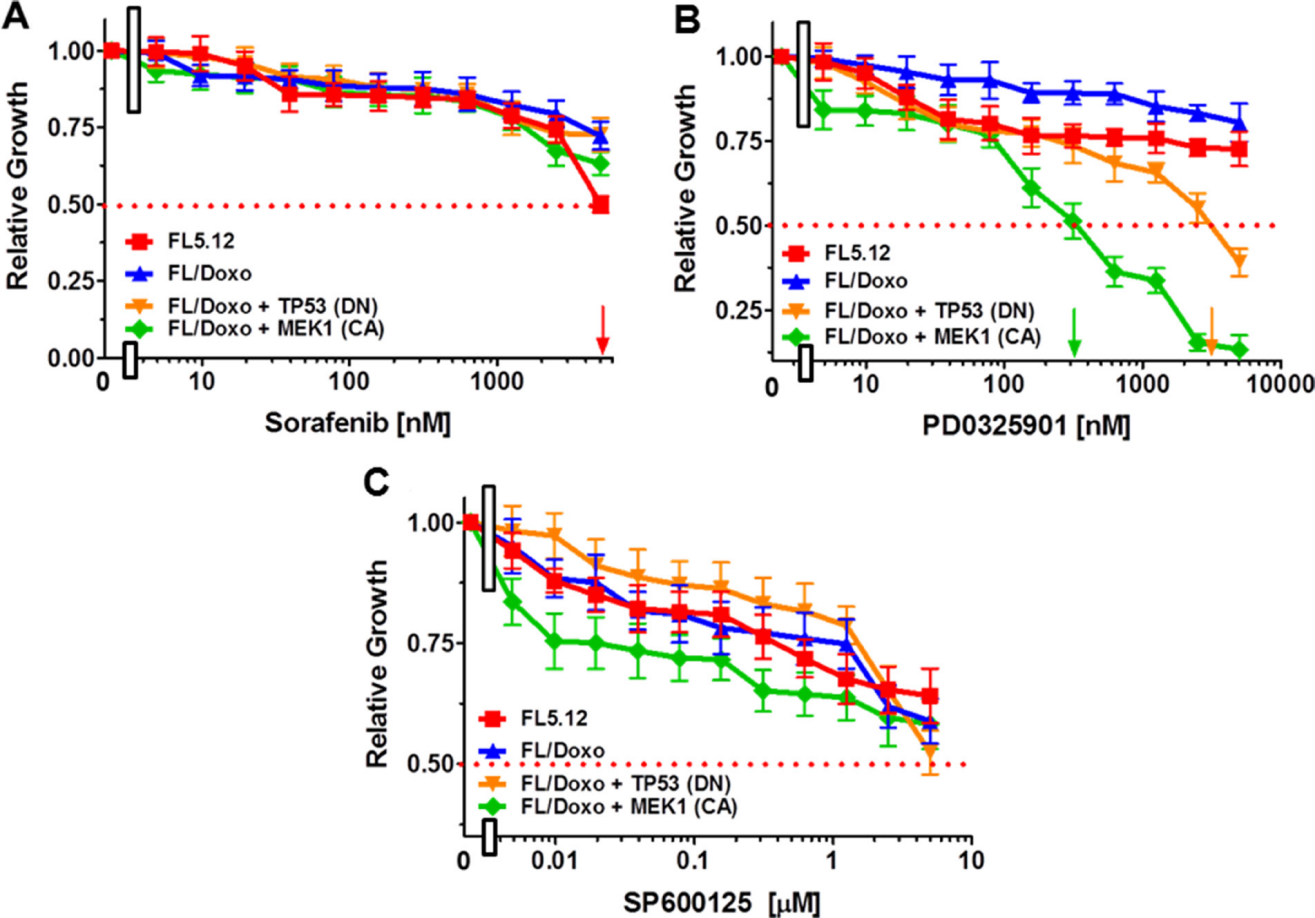
Effects of the Multi-Kinase Inhibitor Sorafenib, MEK1 (PD0325901) and JNK Inhibitors on FL5.12, FL/Doxo, FL/Doxo + TP53 (DN) and FL/Doxo + MEK1(CA) Cells The effects of these inhibitors on: FL5.12 (solid red squares), FL/Doxo (solid blue upward triangles), FL/Doxo + TP53 (DN) (solid orange downward diamonds) and FL/Doxo + MEK1 (CA) (solid green diamonds) cells were determined by titrating all of the cells on the same day with: Sorafenib (Panel **A**), PD0329501 (Panel **B**) and SP600125 (Panel **C)** inhibitors and then examined four days later by MTT analysis as described [21]. Arrows pointing to the X-axis indicate where the IC_50_ can be estimated. Statistical analysis (unpaired *t* test results) indicated that the two-tailed *P* values for FL/Doxo + MEK1(CA) and FL/Doxo + TP53 (DN) vs FL/Doxo in Panel B was less than 0.0001 which is considered to be extremely statistically significant. These experiments were performed three times with similar results.

The RAF/MEK/ERK pathway has been shown to be involved in the cytokine-dependency and drug resistance of various types of cells (*e.g*., breast, hematopoietic, liver, prostate and other cancers) [9, 12–14, 25, 76]. The effects of treatment of these cells with RAF, MEK or JNK inhibitors were examined (Figure 2). Sorafenib is a small molecule multi-kinase inhibitor which targets RAF, FLT3 and cytokine-receptors [*e.g*., platelet derived growth factor receptor (PDGFR)] [74–79]. These cells were relatively resistant to the sorafenib inhibitor. Concentrations of greater than 5 μM sorafenib were required to reach the IC_50_ of FL/Doxo, FL/Doxo + MEK1 (CA) and FL/Doxo + TP53 (DN) cells. An IC_50_ of approximately 5 μM was observed with FL5.12 cells (Figure 2A).

In contrast, the FL/Doxo + MEK1 (CA) and FL/Doxo + TP53 (DN) were more sensitive to the MEK1 inhibitor PD0325901 than the FL5.12 and FL/Doxo cells (Figure 2B). IC_50_s of approximately 300 nM and 3,000 nM were observed with FL/Doxo + MEK1 (CA) and FL/Doxo + TP53 (DN) cells, respectively, while concentrations of greater than 5 μM were required to reach the IC_50_ of FL/Doxo and FL5.12 cells. Interestingly, introduction of the MEK1 (CA) into FL/Doxo cells [FL/Doxo + MEK1 (CA)] conferred sensitivity to the MEK inhibitor.

The effects of treatment with the JNK inhibitor SP600125 were examined. In general, all cells were not very sensitive to this inhibitor, as concentrations of greater than 5 μM were required to reach the IC_50_ with the exception of the FL/Doxo + TP53 (DN) cells where an IC_50_ of approximately 5 μM was observed (Figure 2C).

### Differential sensitivity to PI3K/AKT/mTORC1 and BCL2/BCLXL inhibitors

**Figure 3:**
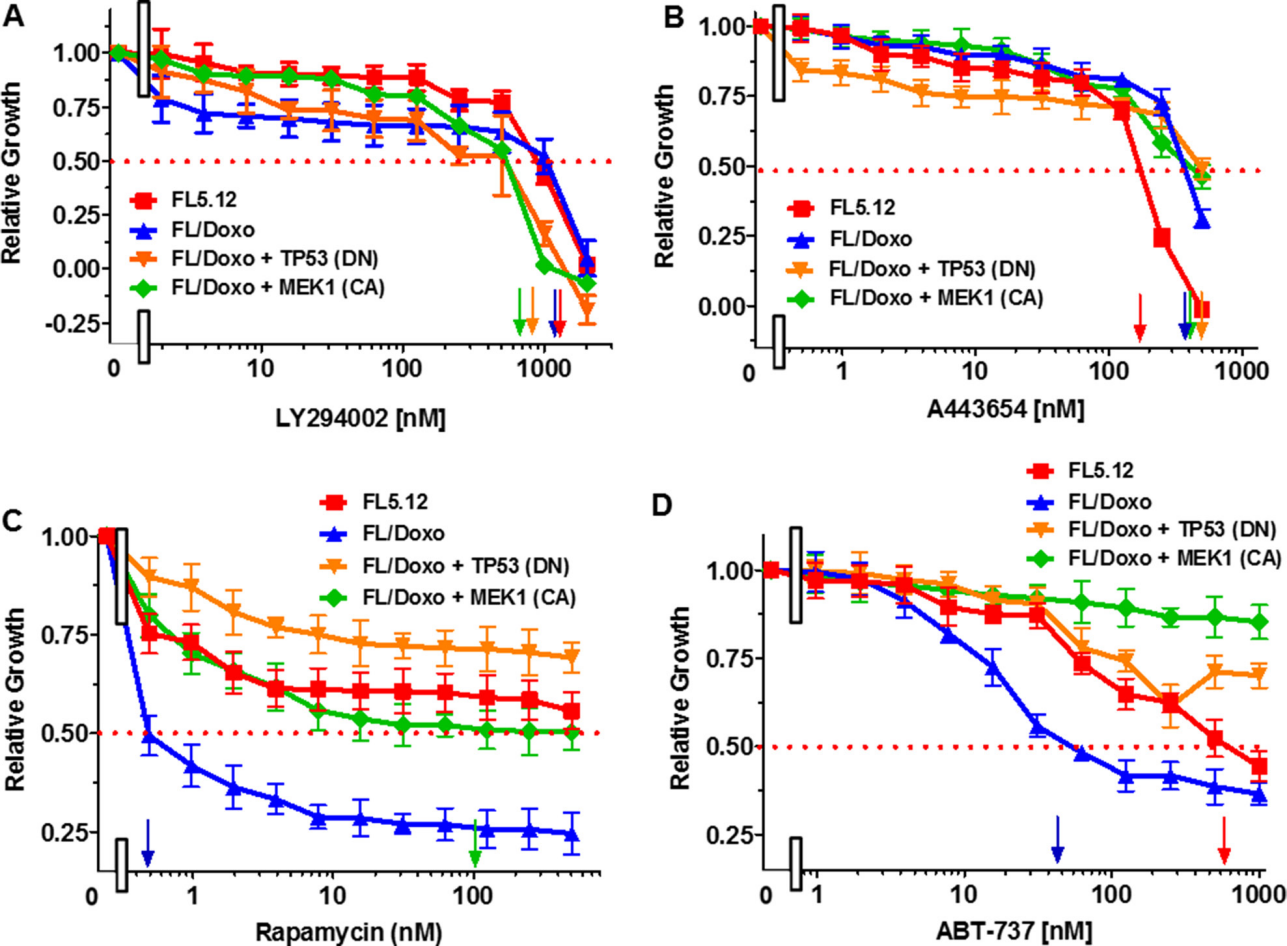
Effects of the PI3K Inhibitor LY294002, Akt Inhibitor A443654, mTORC1 blocker Rapamycin and BCL2 Inhibitor ABT-737 on: FL5.12, FL/Doxo, FL/Doxo + TP53 (DN) and FL/Doxo + MEK1(CA) Cells The effects of these inhibitors onFL5.12 (solid red squares), FL/Doxo (solid blue upward triangles), FL/Doxo + TP53 (DN) (solid orange downward diamonds) and FL/Doxo + MEK1(CA) (solid green diamonds) cells were determined by titrating all of the cells on the same day with: LY294002 (Panel **A**), A443654 (Panel **B**), Rapamycin (Panel **C**) and ABT-737 (Panel **D**) inhibitors and then examined four days later by MTT analysis as described [21]. Arrows pointing to the X-axis indicate where the IC50 can be estimated. Statistical analysis (unpaired *t* test results) indicated that the two-tailed *P* values for FL5.12 or FL/Doxo vs either FL/Doxo + MEK1(CA) or FL/Doxo + TP53 (DN) in Panel A were 0.0030 which is considered very statistically significant. In Panel B, the *P* values between FL5.12 and either FL/Doxo, FL/Doxo + TP53 (DN), or FL/Doxo + MEK1 (CA) were determined to be less than 0.0001 which is considered to be extremely highly significant. In Panel C, the *P* value between FL/Doxo and FL/Doxo + MEK1 (CA) was determined to be less than 0.0001 which is considered to be extremely highly significant. In Panel D, the *P* value between FL/Doxo and FL5.12 was determined to be less than 0.0001 which is considered to be extremely highly significant. These experiments were performed three times with similar results.

We and others have also demonstrated that the PI3K/PTEN/AKT/mTORC1 pathway is involved in drug-resistance of various cancer types and abrogation of cytokine- dependence of hematopoietic cells [5, 6, 10–12, 16, 20, 24, 27–30, 33, 35, 36, 38–41, 43–55, 60, 63, 65, 67, 80–82]. The effects of targeting the PI3K/PTEN/Akt/mTORC1 pathway were also examined (Figure 3). Treatment of the cells with the PI3K inhibitor LY294002 resulted in the IC_50_s of 1,200 nM, 1,200 nM, 500 nM and 500 nM for FL5.12, FL/Doxo, FL/Doxo + TP53 (DN) or FL/Doxo + MEK1 (CA) cells respectively (Figure 3A). Thus, the two lines that were more doxorubicin-resistant [FL/Doxo + TP53 (DN) and FL/Doxo + MEK1 (CA)] were more sensitive than FL5.12 and FL/Doxo cells to the PI3K inhibitor, LY294002.

The effects of treatment with the AKT inhibitor A443654 were examined. IC_50_s of 200 nM and 500 nM were observed with FL5.12 and FL/Doxo respectively. In contrast, greater than 500 nM A443654 were required to reach the IC_50_ of FL/Doxo + TP53 (DN) and FL/Doxo + MEK1 (CA) cells.

The sensitivities of these cells to the mTORC1 blocker rapamycin were examined (Figure 3C). Interestingly, the FL/Doxo line was very sensitive to the mTORC1 blocker rapamycin and an IC_50_ of approximately 0.5 nM was observed (Figure 3C). An IC_50_ of approximately 15 nM rapamycin was observed with the FL/Doxo + MEK1(CA) cells. In contrast, the FL5.12 and FL/Doxo + TP53 (DN) cells were not as sensitive to rapamycin and the IC_50_ for these two lines was not reached with up to 500 nM doxorubicin. The FL/Doxo + TP53 (DN) cells were not very sensitive to rapamycin at these concentrations. These studies point to the importance of mTORC1 in the growth of the drug-resistant FL/Doxo cells and introduction of TP53 (DN) suppressed the effects of rapamycin on these cells.

The sensitivities of the cells to the BCL2 inhibitor ABT-737 were determined (Figure 3D). Interestingly, the FL/Doxo cells were more sensitive to this inhibitor and an IC_50_ of approximately 55 nM was observed. In contrast, an IC_50_ of approximately 600 nM was observed with FL5.12 cells. The FL/Doxo + TP53 (DN) and FL/Doxo + MEK1 (CA) cells were less sensitive to the ABT-737 inhibitor.

### Combining MG-132 and doxorubicin to increase therapeutic effectiveness

**Figure 4:**
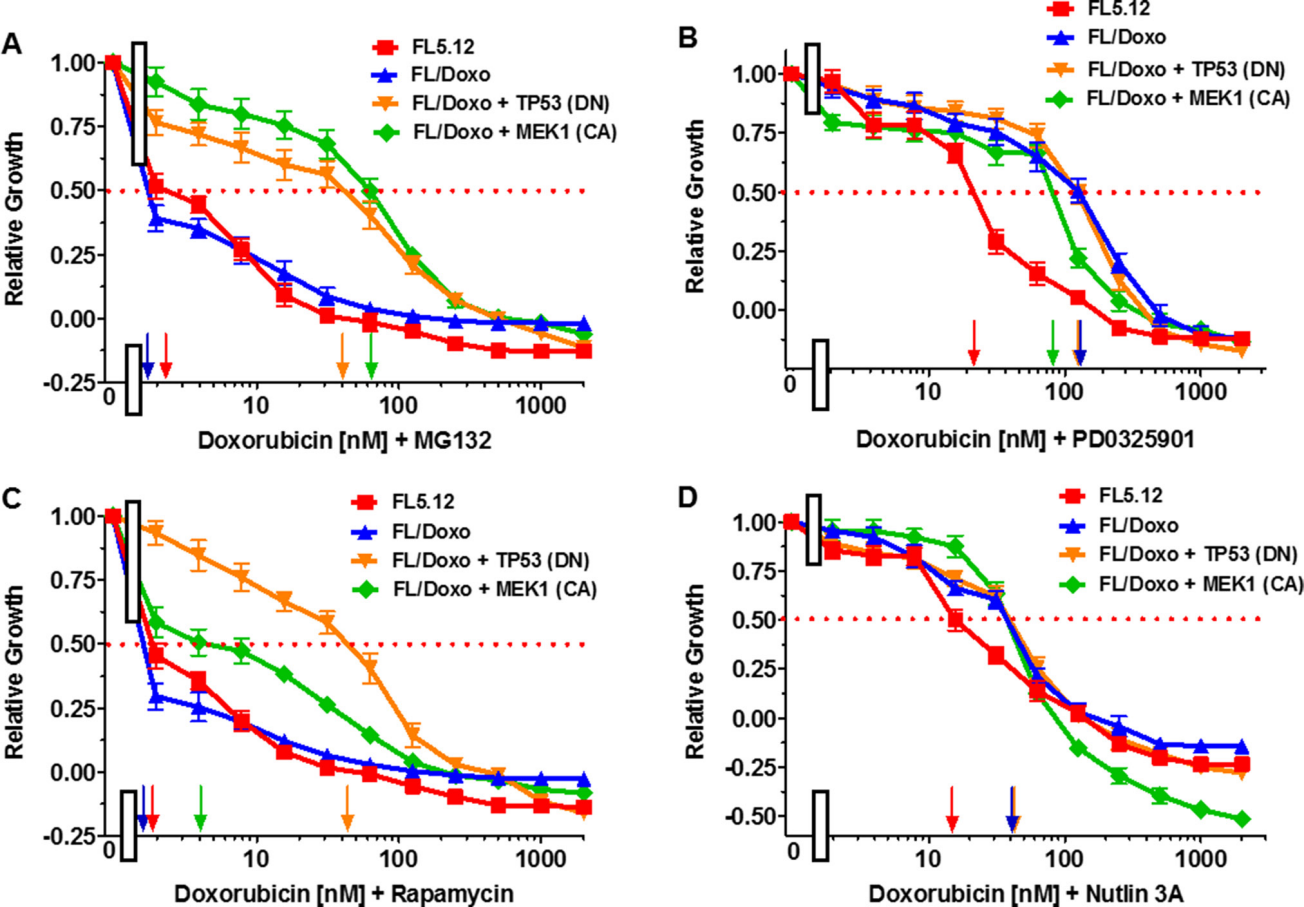
Effects of Different Doses of Doxorubicin in Combination with Suboptimal Constant Doses of MG-132, PD0325901, Rapamycin and Nutlin-3a on: FL5.12, FL/Doxo, FL/Doxo + TP53 (DN) and FL/Doxo + MEK1 (CA) Cells The effects of these inhibitors onFL5.12 (solid red squares), FL/Doxo (solid blue upward triangles), FL/Doxo + TP53 (DN) (solid orange downward diamonds) and FL/Doxo + MEK1(CA) (solid green diamonds) cells were determined by titrating all of the cells on the same day with doxorubicin and suboptimal doses of 10 nM MG132 (Panel **A**), 500 nM PD0325901 (Panel **B**), 10 nM Rapamycin (Panel **C**) and 500 nM Nutlin-3a (Panel **D**) inhibitors and then examined four days later by MTT analysis as described [21]. Arrows pointing to the X-axis indicate where the IC50 can be estimated. Statistical analysis (unpaired *t* test results) indicated that the two-tailed *P* values for FL5.12 or FL/Doxo vs either FL/Doxo + MEK1(CA) or FL/Doxo + TP53 (DN) in Panel A were less than 0.0001 which is considered extremely statistically significant. In Panel B, the *P* values between FL5.12 and either FL/Doxo, FL/Doxo + TP53 (DN), or FL/Doxo + MEK1 (CA) were determined to be less than 0.0001 which is considered to be extremely highly significant. In Panel C, the *P* values between FL/Doxo + TP53 or FL/Doxo + MEK1 (CA) vs FL5.12 or FL/Doxo weredetermined to be less than 0.0001 which is considered to be extremely highly significant. In Panel D, the *P* values between FL5.12 vs FL/Doxo, FL/Doxo + TP53 (DN) or FL/Doxo + MEK1 (CA) were determined to be less than 0.0001 which is considered to be extremely highly significant. These experiments were performed four times with similar results.

The effects of combining MG-132 with doxorubicin were examined (Figure 4A). The cells were also titrated for their sensitivity to doxorubicin in these same experiments. Doxorubicin IC_50_s of approximately 10 nM, 25 nM, 100 nM and 300 nM were observed in these experiments with FL5.12, FL/Doxo, FL/Doxo + TP53 (DN) and FL/Doxo + MEK1 (CA) cells. Addition of the suboptimal dose of MG-132 (10 nM) reduced the IC_50_ for doxorubicin approximately 5-fold in FL5.12 cells to approximately 2 nM, >50-fold in FL/Doxo cells to < 2 nM, 2.3-fold to 45 nM in FL/Doxo + TP53 (DN) and 4.3-fold to 70 nM in FL/Doxo + MEK1 (CA) cells (Figure 4A). Thus, treatment with MG-132 reduced the resistance of the cells to doxorubicin, more significantly in the FL/Doxo, FL5.12 and FL/Doxo + MEK1 (CA) cells than in FL/Doxo + TP53 (WT) cells. These experiments point to the importance of the proteasome in the sensitivity to doxorubicin, especially in the cells that were more sensitive to doxorubicin. Furthermore, the cells with TP53 (DN) were less sensitive to the combination of doxorubicin and MG-132.

### Combining PD0325901 and doxorubicin to increase therapeutic effectiveness

The effects of combining PD0325901 with doxorubicin were examined (Figure 4B). The cells were also titrated for their sensitivity to doxorubicin in these same experiments. Doxorubicin IC_50_s of approximately 40 nM, 100 nM, 120 nM and 300 nM were observed in these experiments for: FL5.12, FL/Doxo, FL/Doxo + TP53 (DN) and FL/Doxo + MEK1 (CA) cells. Addition of the suboptimal dose of PD0325901 (100 nM) reduced the IC_50_ for doxorubicin approximately: 3.3-fold in FL5.12, 3-fold in FL/Doxo + MEK1 (CA) cells, and 1.5-fold FL/Doxo + TP53 (DN) cells but not in FL/Doxo cells (Figure 4B). These experiments point to the importance of the MEK1 in the sensitivity of FL5.12 and FL/Doxo + MEK1 (CA) cells to doxorubicin.

### Combining rapamycin and doxorubicin to increase therapeutic effectiveness

The effects of treatment of the cells with doxorubicin and a suboptimal does of rapamycin were determined (Figure 4C). The cells were also titrated for their sensitivity to doxorubicin in these same experiments. Doxorubicin IC_50_s of approximately: 12 nM, 22 nM, 90 nM and 320 nM were observed in these experiments for FL5.12, FL/Doxo, FL/Doxo + TP53 (DN) and FL/Doxo + MEK1 (CA) cells. Addition of a suboptimal dose of rapamycin (10 nM) reduced the IC_50_ for doxorubicin approximately 6-fold in FL5.12 cells to approximately 2 nM, > 11-fold in FL/Doxo cells to < 2 nM, 2-fold to 42 nM in FL/Doxo + TP53 (DN) and 80-fold in FL/Doxo + MEK1 (CA) cells to approximately 4 nM. Thus, treatment with rapamycin reduced the resistance of the cells to doxorubicin, more significantly in the FL/Doxo + MEK1 (CA) than FL/Doxo or FL5.12 cells. There was only a 2-fold reduction in FL/Doxo + TP53 (WT) cells. These experiments point to the importance of the mTORC1 in sensitivity to doxorubicin and point to an important interaction between the MEK and mTORC1 pathways in drug-resistance.

### Combining nutlin-3a and doxorubicin to increase therapeutic effectiveness

The effects of treatment of the cells with doxorubicin and a suboptimal dose of nutlin-3a were determined (Figure 4D). The cells were also titrated for their sensitivity to doxorubicin in these same experiments. Doxorubicin IC_50_s of approximately 12 nM, 100 nM, 210 nM and 300 nM were observed in these experiments for FL5.12, FL/Doxo, FL/Doxo + TP53 (DN) and FL/Doxo + MEK1 (CA) cells. Addition of the suboptimal dose of nutlin-3a (500 nM) reduced the IC_50_ for doxorubicin approximately: 6-fold in FL5.12 cells to < 2 nM, 50-fold in FL/Doxo cells to < 2 nM and 5-fold to 42 nM in FL/Doxo + TP53 (DN) and 75-fold in FL/Doxo + MEK1 (CA) cells to 4 nM. Thus, treatment with nutlin-3a reduced the resistance of the cells to doxorubicin, more significantly in the FL/Doxo + MEK1 (CA), FL/Doxo and FL5.12 cells than in cells containing DN TP53 gene. However, these results also demonstrated that in the FL/Doxo + TP53 (DN) that still have endogenous WT TP53 genes, there is some sensitivity to the nutlin-3a MDM2 inhibitor.

### Combining ABT-737 and doxorubicin or the PI3K inhibitor LY294002 to increase therapeutic effectiveness

**Figure 5:**
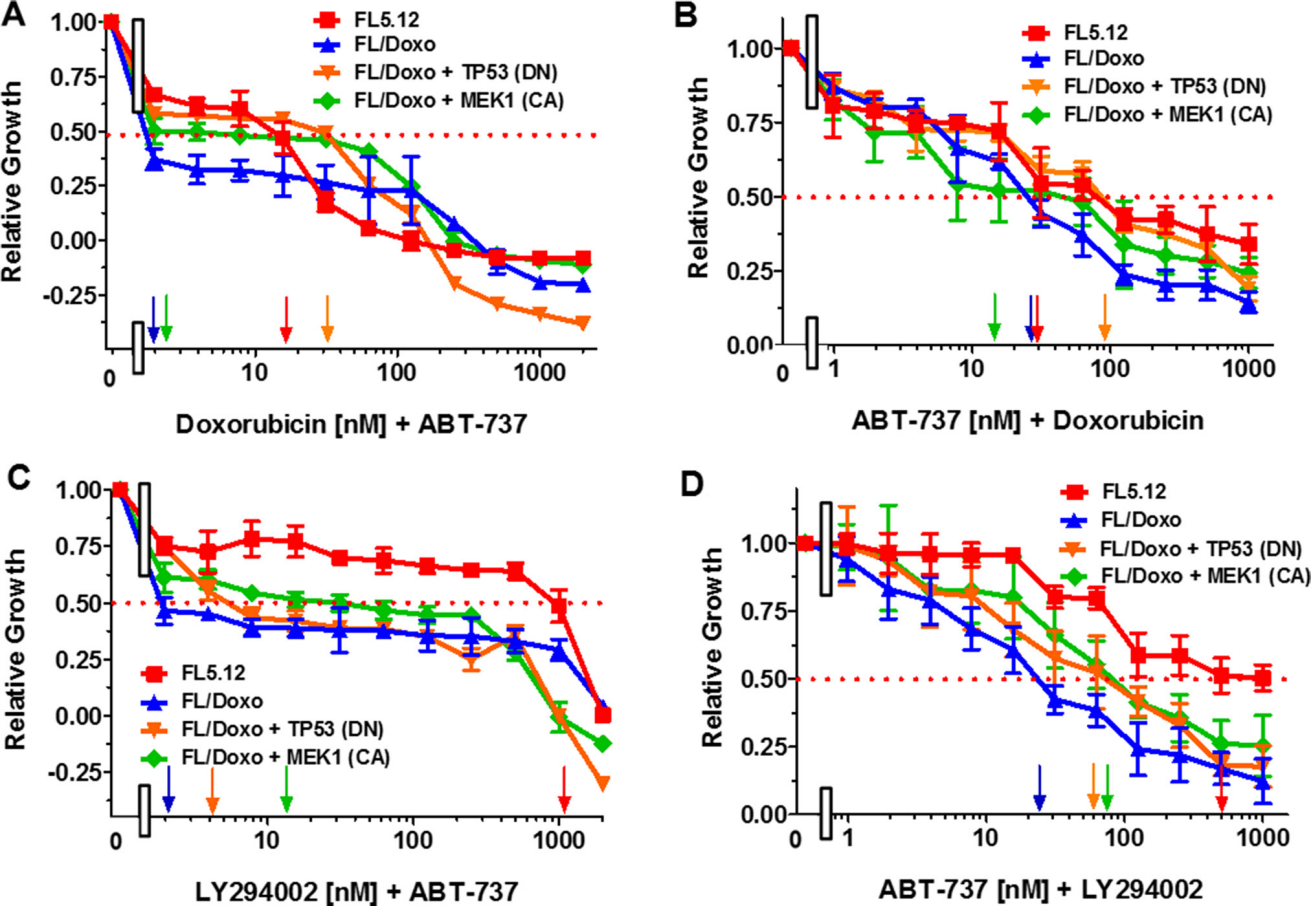
Effects of different doses of ABT-737 in combination with Doxorubicin or the PI3K Inhibitor LY294002 The effects of these inhibitors on: FL5.12 (solid red squares), FL/Doxo (solid blue upward triangles), FL/Doxo + TP53 (DN) (solid orange downward diamonds) and FL/Doxo + MEK1(CA) (solid green diamonds) cells were determined by titrating all of the cells with doxorubicin on the same day and a suboptimal dose of 500 nM ABT-737 (Panel **A**), or ABT-737 and a suboptimal dose of 5 nM doxorubicin and then examined four days later by MTT analysis as described [21]. The effects of LY294002 or ABT-737 inhibitors on: FL5.12 (solid red squares), FL/Doxo (solid blue upward triangles), FL/Doxo + TP53 (DN) (solid orange downward diamonds) and FL/Doxo + MEK1(CA) (solid green diamonds) cells were determined by titrating all of the cells on the same day with LY294002 and a suboptimal dose of 500 nM ABT-737 (Panel **C**), or ABT-737 and a suboptimal dose of 500 nM LY294002 (Panel **D**). Arrows pointing to the X-axis indicate where the IC_50_ can be estimated. Statistical analysis (unpaired *t* test results) indicated that the two-tailed *P* values for FL5.12 or FL/Doxo + TP53 (DN) vs FL/Doxo or FL/Doxo + MEK1 (CA) in Panel A were 0.0001 which is considered extremely statistically significant. In Panel **B**, the *P* values between FL/Doxo + TP53 (DN) vs FL5.12, FL/Doxo or FL/Doxo + MEK1 (CA) were determined to be less than 0.0001 which is considered to be extremely highly significant. In this same panel, the *P* values between FL5.12 or FL/Doxo vs FL/Doxo +MEK1 (CA) were determined to equal 0.0002 which is considered to be extremely statistically significant. In Panel C, the *P* values between FL5.12 vs FL/Doxo, FL/Doxo + TP53 (DN) or FL/Doxo + MEK1 (CA) were determined to be less than 0.0001 which is considered to be extremely highly significant. In Panel D, the *P* values between FL5.12 vs FL/Doxo, FL/Doxo + MEK1 (CA) or FL/Doxo + TP53 (DN) were determined to be less than 0.0001 which is considered to be extremely highly significant. In this same panel, the *P* values between FL/Doxo vs FL/Doxo + MEK1 (CA) or FL/Doxo + TP53 (DN) were determined to equal 0.0009 which is considered to be extremely highly significant. These experiments were performed three-times with similar results.

The effects of combining ABT-737 with doxorubicin were examined (Figure 5). The cells were also titrated for their sensitivity to doxorubicin in these same experiments. Doxorubicin IC_50_s of approximately: 15 nM, 60 nM, 90 nM and 300 nM were observed in these experiments for FL5.12, FL/Doxo, FL/Doxo + TP53 (DN) and FL/Doxo + MEK1 (CA) cells. Addition of the suboptimal dose of ABT-737 (50 nM) reduced the IC_50_ for doxorubicin approximately, 30-fold in FL/Doxo, 4-fold FL/Doxo + TP53 (DN) cells and 100-fold in FL/Doxo + MEK1 (CA) cells (Figure 5A).

The effects of combining a suboptimal dose of doxorubicin with ABT-737 were also determined (Figure 5B). The suboptimal dose of doxorubicin reduced the IC_50_ for ABT-737 in all four cell lines ranging from approximately 12 nM in FL/Doxo + MEK1 (CA) to 30 nM in FL/Doxo and FL5.12 cells and to 90 nM in FL/Doxo + TP53 (DN) cells (Figure 5B). These experiments point to the importance of the BCL2/BCLXL in the sensitivity of all the cells to doxorubicin. Furthermore, these experiments demonstrate that while the FL/Doxo + MEK1 (CA) cells were not very sensitive to treatment with the BCL2 inhibitor by itself (Figure 3D), but when the BCL2 inhibitor was combined with doxorubicin, the FL/Doxo + MEK1 (CA) cells become very sensitive.

The effects of combining BCL2 and the PI3K inhibitor (LY294002) were also determined. Different concentrations of LY294002 were combined with a suboptimal dose of ABT-737 (50 nM) in these experiments (Figure 5C). Addition of a suboptimal dose of ABT-737 lowered the amount of relative growth in FL/Doxo, FL/Doxo + TP53 (DN) and FL/Doxo + MEK1 (CA). However, the slopes of the dose response lines did not really change.

The effects of different concentrations of ABT-737 and a constant dose of 500 nM LY294002 was determined (Figure 5D). Addition of the suboptimal dose of LY294002 reduced the IC_50_ for ABT-737 approximately 2.2-fold in FL/Doxo cells and >10-fold in FL/Doxo + TP53 (DN) and FL/Doxo + MEK1 (CA) cells. In contrast, addition of the suboptimal dose of LY294002 did not reduce the IC_50_ for ABT-737 in FL5.12 cells.

### Combining ABT-737 and PD0325901 to increase therapeutic effectiveness

**Figure 6:**
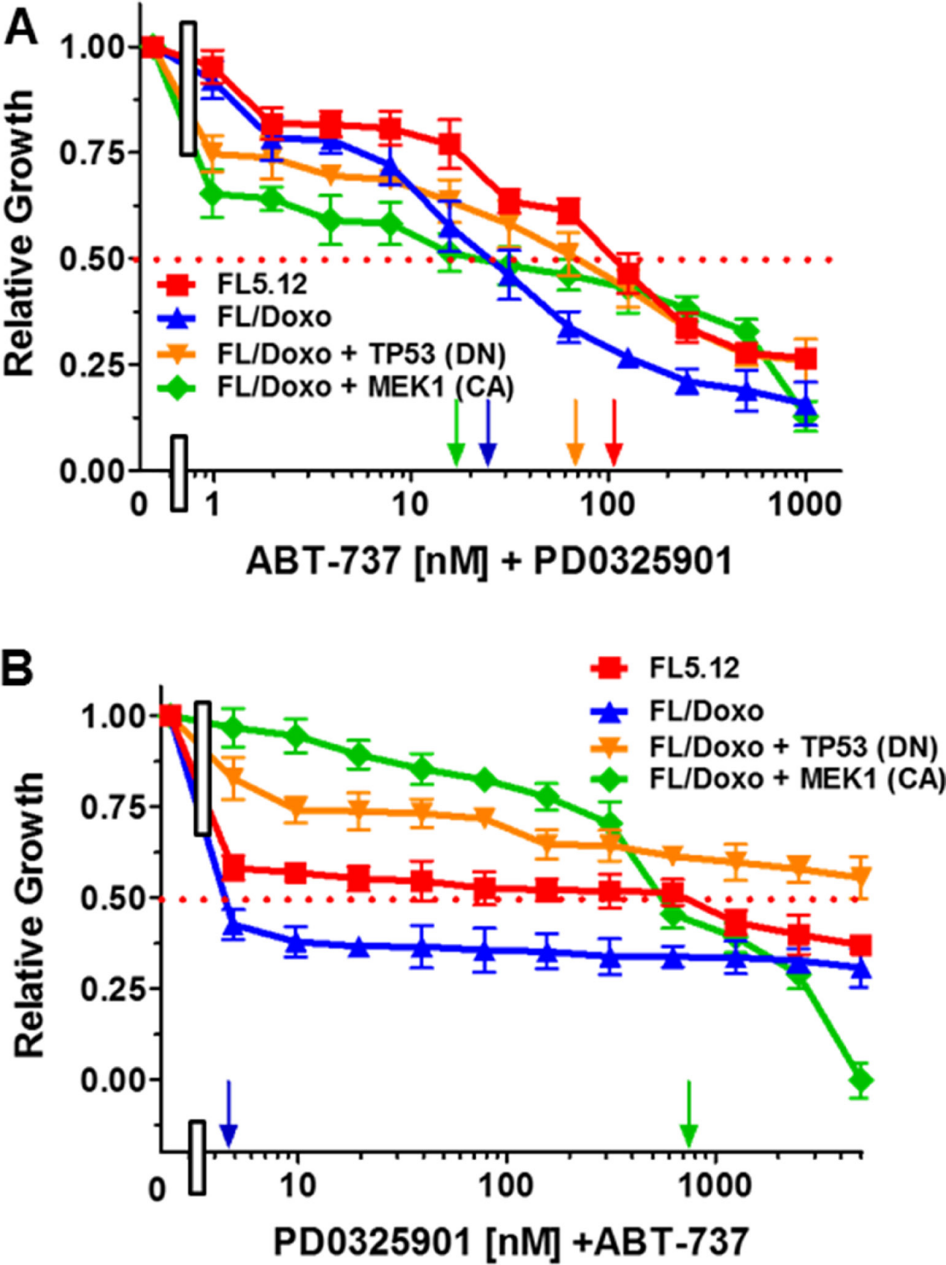
Effects of different doses of ABT-737 or PD0325901 in combination with suboptimal constant doses of PD0325901 or ABT-737 The effects of these inhibitors on:FL5.12 (solid red squares), FL/Doxo (solid blue upward triangles), FL/Doxo + TP53 (DN) (solid orange downward diamonds) and FL/Doxo + MEK1(CA) (solid green diamonds) cells were determined by titrating all of the cells on the same day with ABT-737 and a suboptimal dose of 500 nM PD0329501 (Panel **A**), or PD0325901 and a suboptimal dose of 500 nM ABT-737 and then examined four days later by MTT analysis as described [21]. Arrows pointing to the X-axis indicate where the IC50 can be estimated. Statistical analysis (unpaired *t* test results) indicated that the two-tailed *P* values for FL5.12 or FL/Doxo + TP53 (DN) vs either FL/Doxo or FL/Doxo + MEK1(CA) in Panel A were less 0.0001 than which is considered extremely statistically significant. In Panel **B**, the *P* values between FL/Doxo or FL5.12 and FL/Doxo + MEK1 (CA) were determined to be less than 0.0001 which is considered to be extremely highly significant. These experiments were performed three-times with similar results.

The effects of combining ABT-737 and a suboptimal dose of PD0325901 were examined (Figure 6A). Addition of the suboptimal dose of PD0325901 (500 nM) reduced the IC_50_ for ABT-737 greater than 50-fold in FL/Doxo + MEK1 (CA) cells and approximately 1.7-fold in FL/Doxo +TP53 (DN) and 1.5-fold in FL/Doxo cells but did not reduce the IC_50_ for doxorubicin in FL5.12 cells.

The effects of combining PD0325901 and a suboptimal dose of ABT-737 were examined (Figure 6B). Addition of the suboptimal dose of ABT-737 (50 nM) reduced the IC_50_ for PD0325901 greater than 400-fold in FL/Doxo cells and approximately 67-fold in FL5.12 cells. In contrast, this combination of PD0325901 and ABT-737 did not lower the IC_50_ for PD0325901 in either FL/Doxo + MEK1(CA) or FL/Doxo + TP53 (DN) suggesting that further reduction of BCL2/BCLXL activity did not increase the sensitivity of these two lines to the MEK1 inhibitor.

### Combining rapamycin and either MG132 or nutlin-3a to increase therapeutic effectiveness

**Figure 7:**
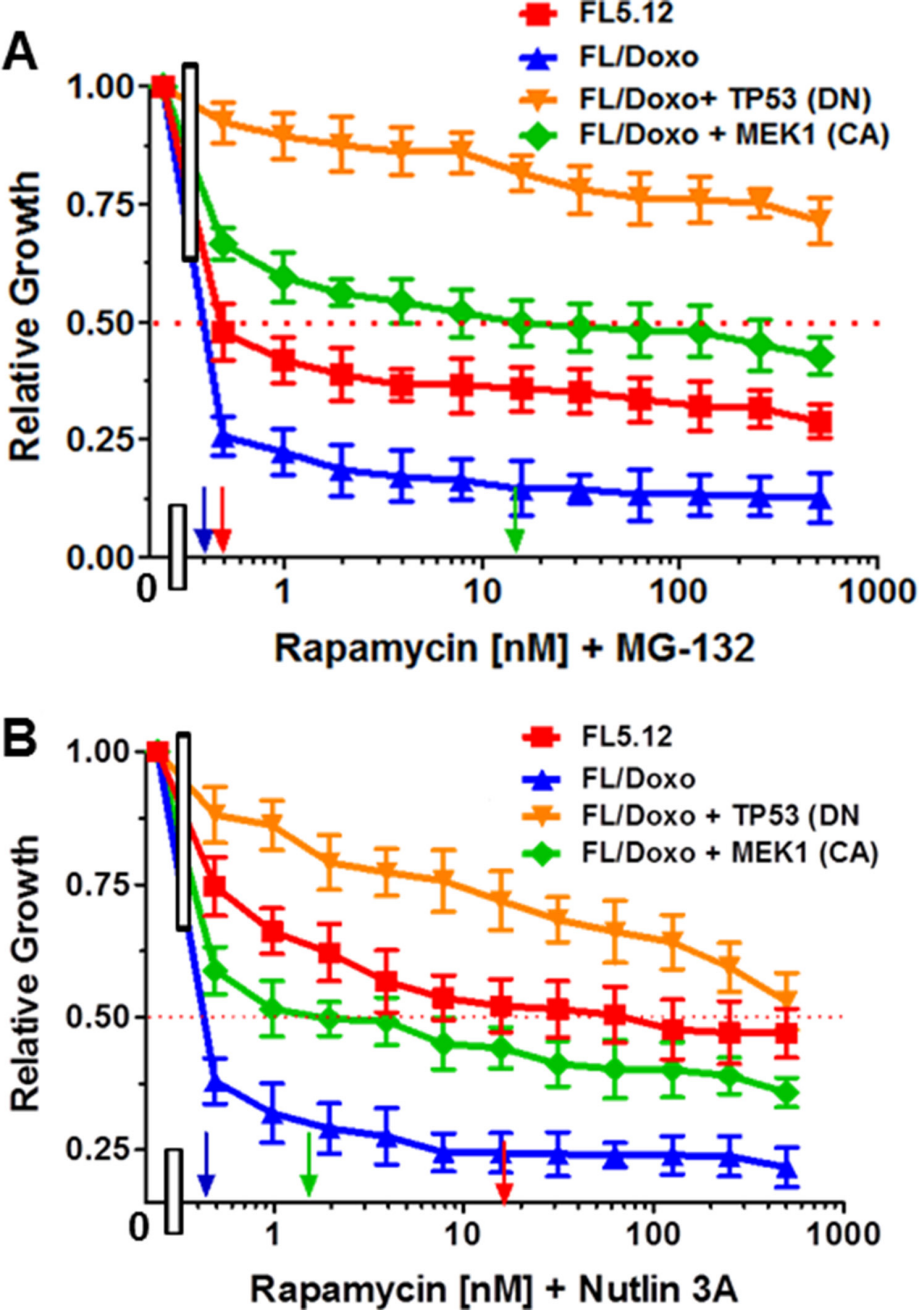
Effects of Different Doses of Rapamycin in Combination with Suboptimal Constant Doses of MG-132 or Nutlin-3a The effects of these inhibitors on: FL5.12 (solid red squares), FL/Doxo (solid blue upward triangles), FL/Doxo + TP53 (DN) (solid orange downward diamonds) and FL/Doxo + MEK1(CA) (solid green diamonds) cells were determined by titrating all of the cells on the same day with rapamycin and a suboptimal dose of 10 nM MG132 (Panel **A**), or a suboptimal dose of 500 nM Nutlin-3a and then examined four days later by MTT analysis as described [21]. Arrows pointing to the X-axis indicate where the IC50 can be estimated. Statistical analysis (unpaired *t* test results) indicated that the two-tailed *P* values for FL5.12 or FL/Doxo vs FL/Doxo + MEK1(CA) in Panel A were less than 0.0001 which are considered extremely statistically significant. In Panel **B**, the *P* values between FL/Doxo + TP53 (DN) vs FL5.12, FL/Doxo FL/Doxo + MEK1 (CA) were determined to be less than 0.0001 which are considered to be extremely highly significant. These experiments were performed three-times with similar results.

The effects of treatment of the cells with rapamycin and a suboptimal does of MG132 were determined (Figure 7A). Addition of the suboptimal dose of MG132 (10 nM) reduced the IC_50_ for rapamycin > 500-fold in FL5.12 cells to approximately 0.5 nM, > 2-fold in FL/Doxo cells to < 2 nM. The addition of MG132 had less effects on the IC_50_ for rapamycin in FL/Doxo + TP53 (DN) and FL/Doxo + MEK1 (CA) cells.

The effects of treatment of the cells with rapamycin and a suboptimal dose of nutlin-3a were determined (Figure 7B). Addition of the suboptimal dose of nutlin-3a (500 nM) reduced the IC_50_ for rapamycin 28-fold in FL5.12 cells to approximately 18 nM, > 2-fold in FL/Doxo cells to < 2 nM. The addition of nutlin-3a reduced the IC_50_ for rapamycin approximately 10-fold to 1 nM in FL/Doxo + MEK1 (CA) cells and had some effects on the IC_50_ for rapamycin in FL/Doxo + TP53 (DN). Thus, the mTORC pathway is important in the drug resistance of these hematopoietic cells.

### Expression of TP53 and signaling proteins in FL5.12, FL/Doxo, FL/Doxo + TP53 (WT) and FL/Doxo + MEK1 (CA) cells

Previously, we determined that when FL5.12 were deprived of the required cytokine IL-3 for 24 hrs., they express TP53 which can be stabilized after treatment with the proteasomal inhibitor MG132 [21]. The levels of TP53 can be increased in these cells upon treatment with 100 and 1,000 nM doxorubicin. In contrast, FL/Doxo cells did not express as much TP53 when they are deprived of IL-3 for 24 hours. However, up addition of 100 or 1,000 nM doxorubicin, there were increases in the levels of TP53 detected [21].

**Figure 8:**
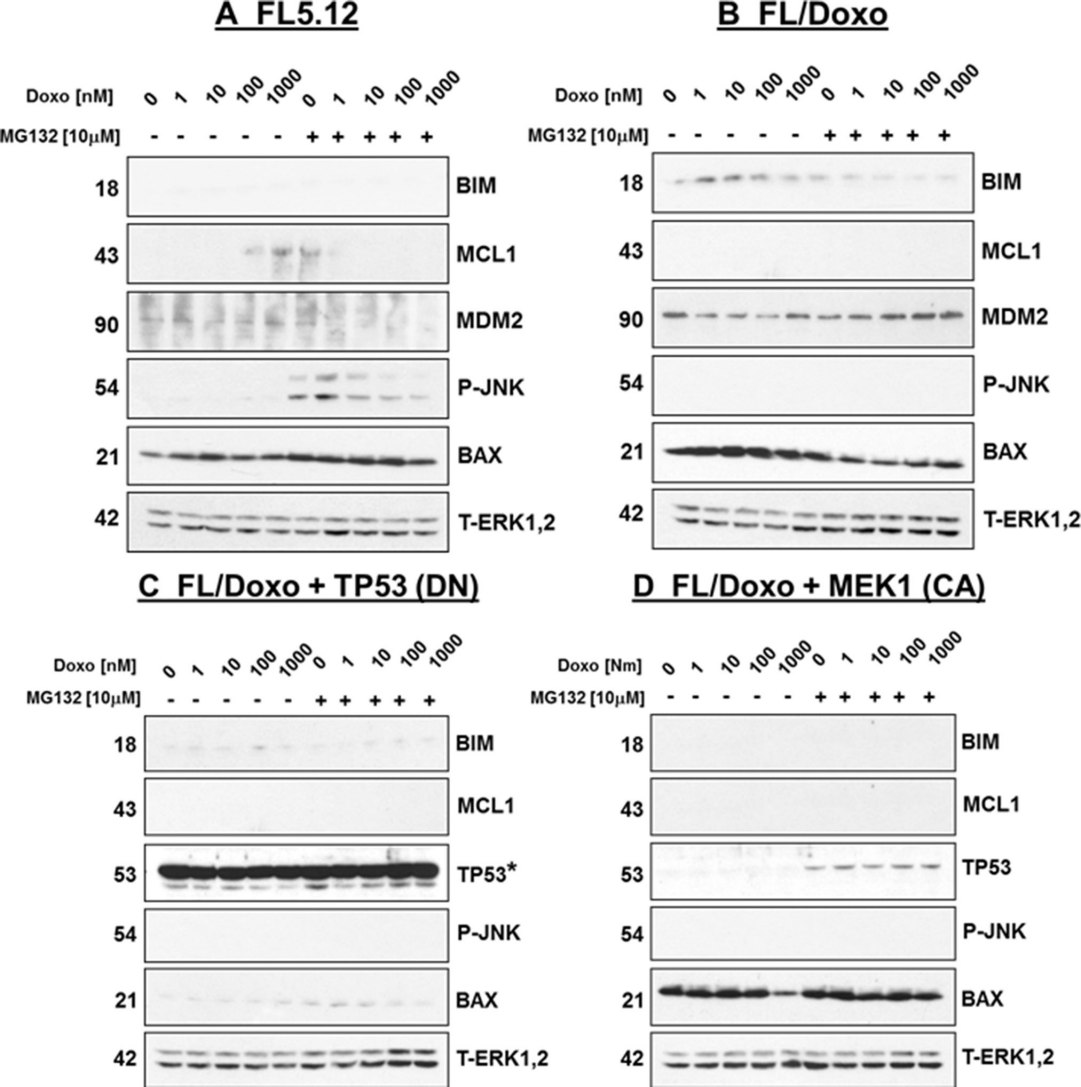
Western blot Analysis of Apoptotic Regulatory Gene Expression, MDM2 and Activated JNK in FL5.12, FL/Doxo,FL/Doxo + TP53 (DN) and FL/Doxo + MEK1 (CA) Cells The effects of treatment with different concentrations of doxorubicin in the absence and presence of 10 μM MG132 was determined as described [21]. The western blots were probed with antibodies to the indicated proteins. * = presence of TP53 (DN) protein.

In the present studies, we determined that no detectable levels of BIM were observed in FL5.12 cells when they were deprived of IL-3 and then treated with doxorubicin or doxorubicin and the MG132 proteasomal inhibitor (Figure 8). In contrast, BIM was detected in FL/Doxo cells when they were IL3-deprived and then treated with doxorubicin but not when they were treated with MG132 and greater than 10 nM doxorubicin, indicated that suppression of the proteasome was inhibiting the expression of BIM in response to doxorubicin treatment.

The expression of MCL1 was upregulated upon treatment with 100 and 1,000 nM doxorubicin of FL5.12 cells but not FL/Doxo cells. Treatment with 10 μM MG132 by itself resulted in the detection of MCL1 in FL5.12 cells but not FL/Doxo cells. The expression of MCL1 was suppressed by the proteasomal inhibitor (MG132) in the presence of doxorubicin in FL5.12 cells. In contrast, the expression of MCL1 was not detected in the FL/Doxo cells in either the presence or absence of MG132 and doxorubicin.

Very low levels of MDM2 were detected in FL5.12 cells, even in the presence of MG132. The FL/Doxo cells were determined to express higher levels of MDM-2 than FL5.12 cells (Figure 8). In contrast to the expression of BIM in FL/Doxo cells, the levels of MDM2 tended to increase slightly in FL/Doxo cells when they were treated with doxorubicin and MG132. These results suggest that there was more MDM2 activity in FL/Doxo cells than FL5.12 cells.

The expression of phospho-JNK was detected in FL5.12 cells when they were treated with MG132 and either low or no doxorubicin. In contrast, phospho-JNK (P-JNK) was not detected in FL5.12 cells when they were cultured in absence of MG132. No P-JNK was detected in FL/Doxo cells in the presence and absence of doxorubicin and MG132. BAX, which is a TP53-regulated gene, was detected in both FL5.12 and FL/Doxo cells. The levels of total and activated ERK and TP53 in FL5.12 and FL/Doxo cells in these same gels were presented previously [21].

The levels of BIM, MCL1, TP53, P-JNK, BAX and ERK1,2 were examined in FL/Doxo + TP53 (DN) and FL/Doxo+MEK1 (CA) cells (Figure 8). The FL/Doxo+MEK1 (CA) cells did not express BIM, MCL1 or P-JNK upon treatment with doxorubicin, MG132, or combined doses of doxorubicin and MG132 treatment as determined by western blot analysis. The FL/Doxo+TP53 (DN) cells, that contained the TP53 (DN) retrovirus, expressed high levels of TP53 protein as it was stabilized by the introduced truncation in the gene. The FL/Doxo + MEK1 (CA) cells expressed similar levels of TP53 as the FL/Doxo cells [21]. The FL/Doxo + TP53 [DN] cells expressed low to undetectable levels of the TP53-inducible gene BAX, suggesting that TP53 (DN) suppressed its expression. BAX expression was detected in FL/Doxo + MEK1(CA) cells. The levels of total ERK1,2 are presented as a loading control.

### Effects of doxorubicin and inhibitors on gene expression

**Figure 9:**
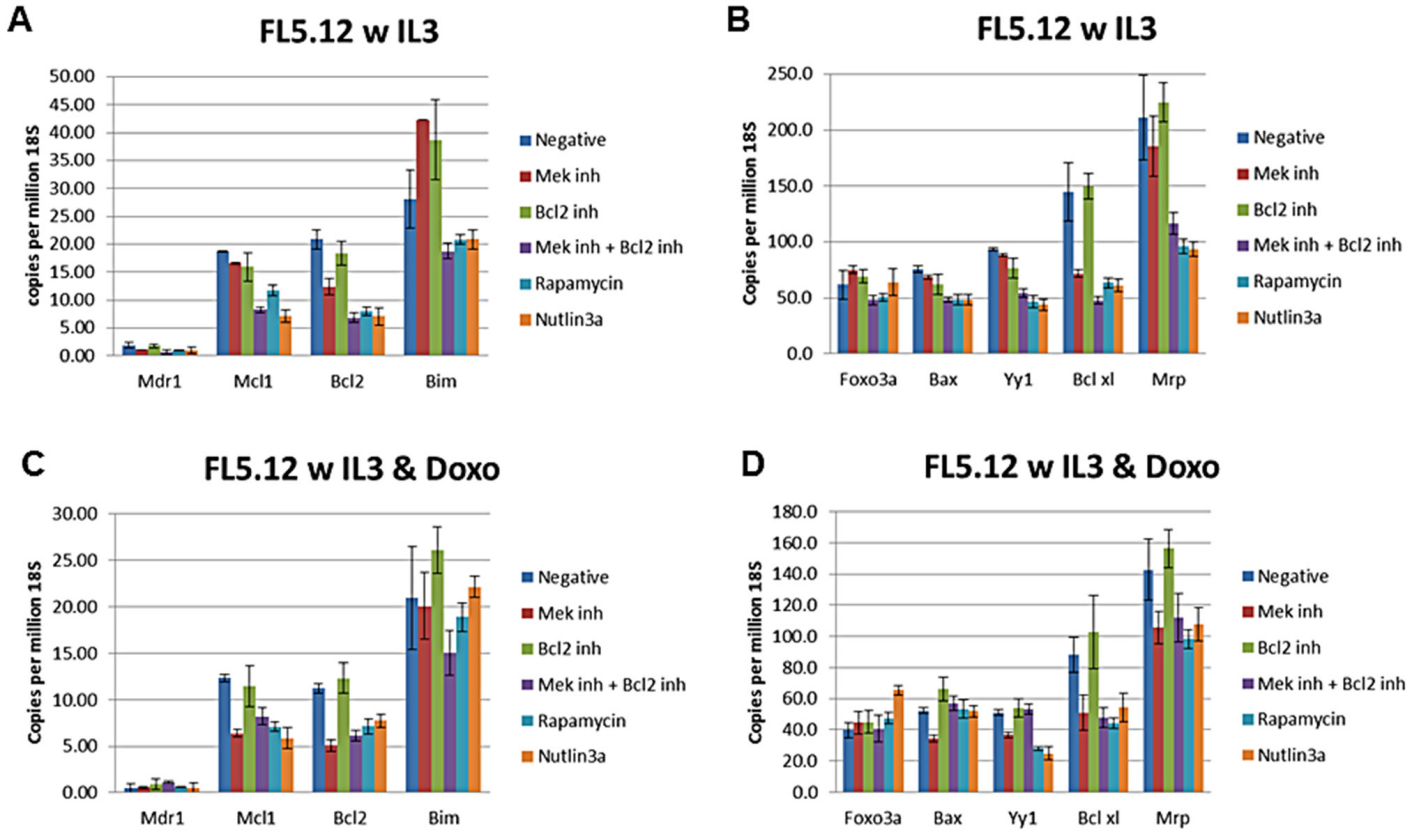
qRT-PCR Analysis of Gene Expression in FL5.12 cells (Panels **A** and **B**) Cells were treated with IL-3 for 24 hours. (Panels **C** and **D**) Cells were treated with IL-3 + 25 nM doxorubicin for 24 hours. The cells were treated with the indicated inhibitors (MEK inhibitor, PD0325901 = 5,000 nM, BCL2 inhibitor ABT-737 = 1000 nM, MEK + BCL2 inhibitors 5,000 nM PD0325901 + 1,000 nM ABT-737 or rapamycin = 100 nM) for 24 hours in medium containing IL-3 and the presence or absence of 25 nM doxorubicin.

**Figure 10:**
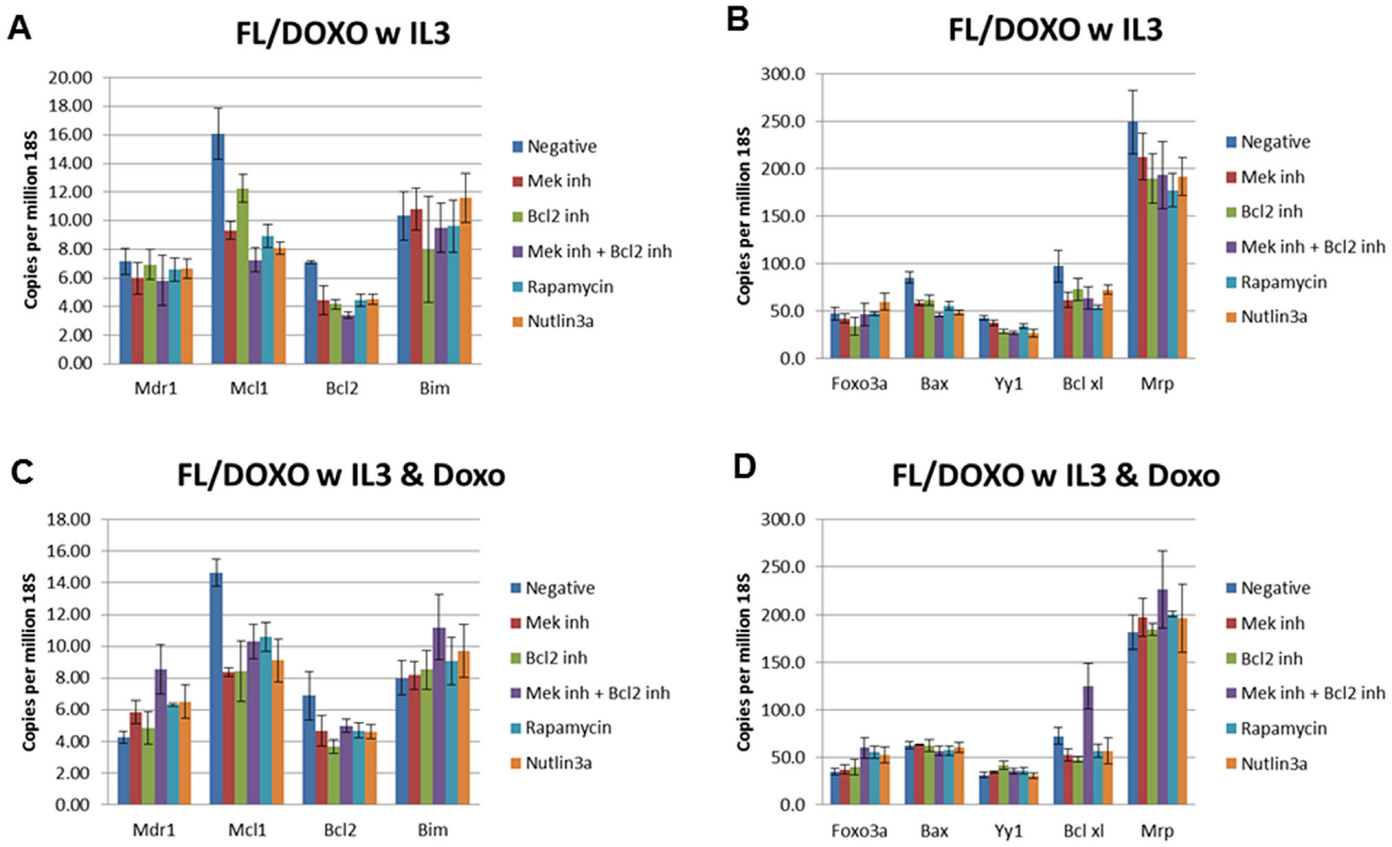
qRT-PCR Analysis of Gene Expression in FL/Doxo cells (Panels **A** and **B**) Cells were treated with IL-3 for 24 hours. (Panels **C** and **D**) Cells were treated with IL-3 + 25 nM doxorubicin for 24 hours. The cells were treated with the indicated inhibitors (MEK inhibitor, PD0325901 = 5,000 nM, BCL2 inhibitor ABT-737 = 1000 nM, MEK + BCL2 inhibitors 5,000 nM PD0325901 + 1,000 nM ABT-737 or rapamycin = 100 nM) for 24 hours in medium containing IL-3 and the presence or absence of 25 nM doxorubicin.

**Figure 11:**
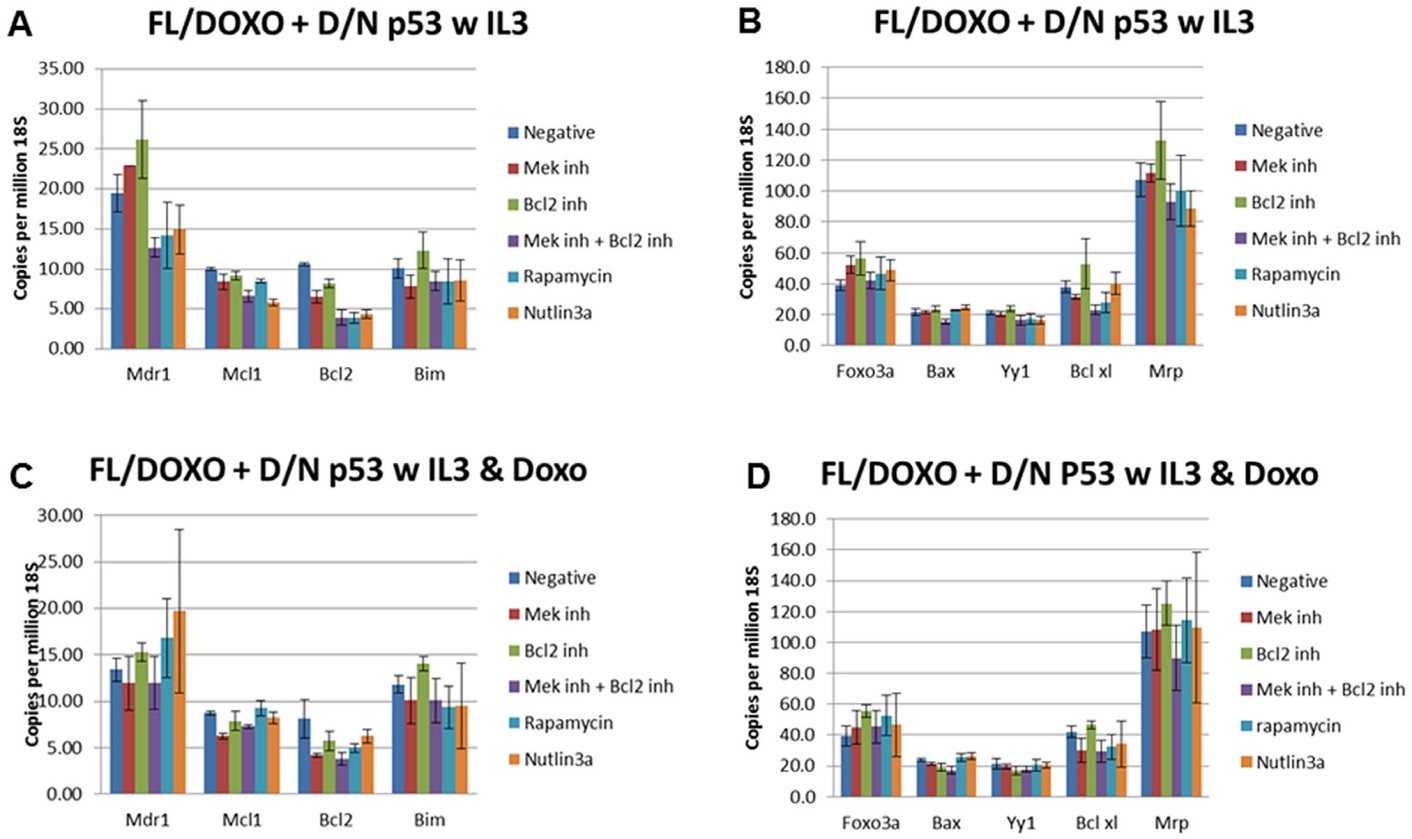
qRT-PCR Analysis of Gene Expression in FL/Doxo + TP53 (DN) cells (Panels **A** and **B**) Cells were treated with IL-3 for 24 hours. (Panels **C** and **D**) Cells were treated with IL-3 + 25 nM doxorubicin for 24 hours. The cells were treated with the indicated inhibitors (MEK inhibitor, PD0325901 = 5,000 nM, BCL2 inhibitor ABT-737 = 1000 nM, MEK + BCL2 inhibitors 5,000 nM PD0325901 + 1,000 nM ABT-737 or rapamycin = 100 nM) for 24 hours in medium containing IL-3 and the presence or absence of 25 nM doxorubicin.

**Figure 12:**
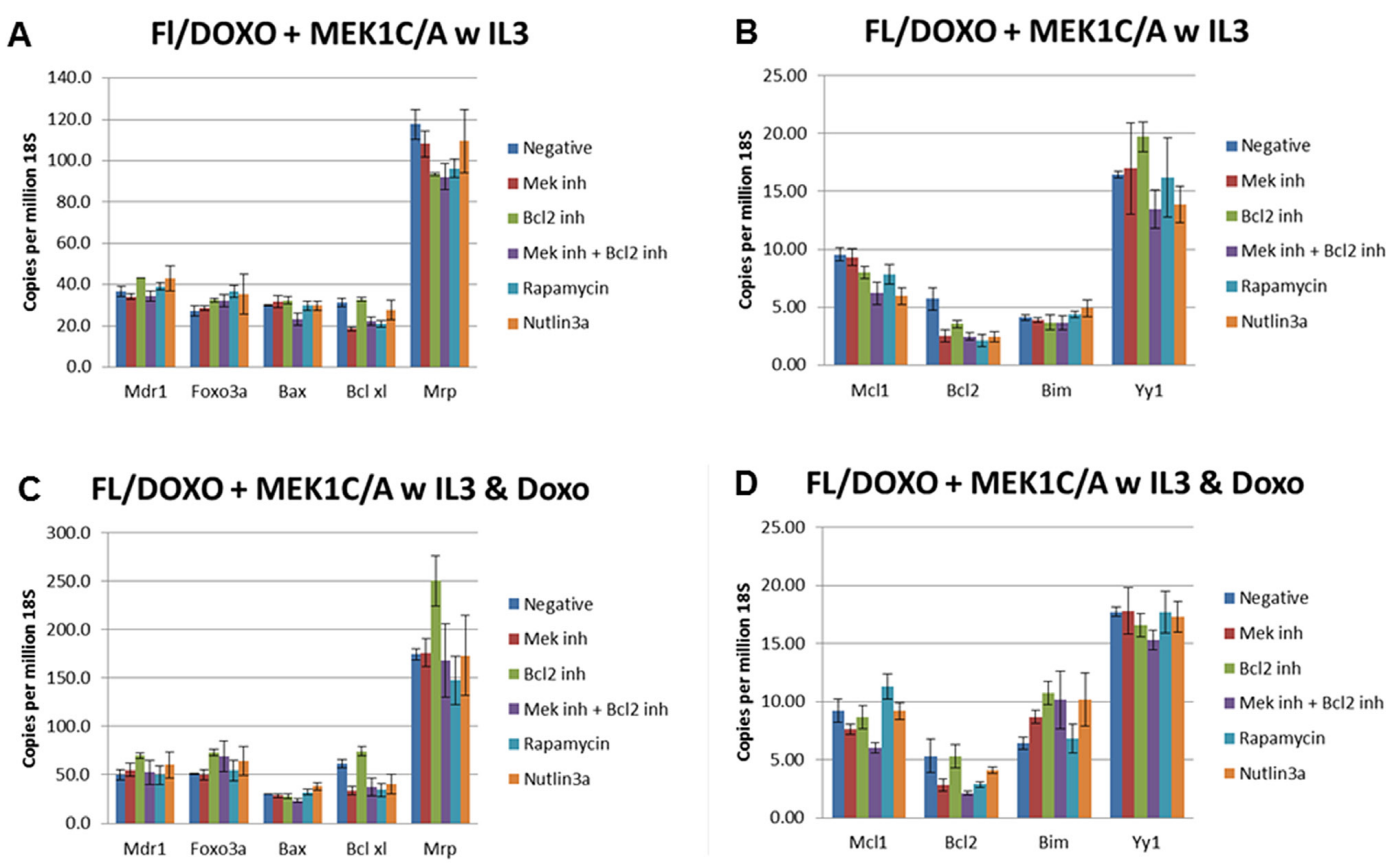
qRT-PCR analysis of gene expression in FL/Doxo + MEK1 (CA) cells (Panels **A** and **B**) Cells were treated with IL-3 for 24 hours. (Panels **C** and **D**) Cells were treated with IL-3 + 25 nM doxorubicin for 24 hours. The cells were treated with the indicated inhibitors (MEK inhibitor, PD0325901 = 5,000 nM, BCL2 inhibitor ABT-737 = 1000 nM, MEK + BCL2 inhibitors 5,000 nM PD0325901 + 1,000 nM ABT-737 or rapamycin = 100 nM) for 24 hours in medium containing IL-3 and the presence or absence of 25 nM doxorubicin.

We examined the expression of some of these and other genes in the presence and absence of doxorubicin by qRT-PCR (Figures 9, 10, 11, 12). In addition, the effects of the MEK (PD0329051), BCL2 (ABT-737), mTORC1 (rapamycin) and MDM2 (nutlin-3a) inhibitors on the expression of key genes were examined by qRT-PCR. The levels of gene expression were normalized to the levels of 18S rRNA molecules detected (Figures 9, 10, 11, 12). We summarize the key differences in gene expression as determined by qRT-PCR between FL5.12, FL/Doxo, FL/Doxo + TP53 (DN) and FL/Doxo + MEK1 (CA) cells.

### Higher levels of MDR1 are detected in doxorubicin-resistant cells

Doxorubicin-sensitive FL5.12 cells expressed low levels of mRNA transcripts encoding MDR1, less than 2 copies (1.91) per million 18S rRNAs (Figure 9). The effects of treatment with the MEK, BCL2, MEK + BCL2, rapamycin and nutlin-3a inhibitors were examined. The biggest decrease in expression of MDR1 (3.1-fold) was observed when FL5.12 cells were treated with both MEK + BCL2 inhibitors as 0.6 copies of MDR1 were detected per million 18S rRNAs. Treatment with the MEK, BCL2, rapamycin or nutlin-3a inhibitors resulted in less than a 2-fold decreases in MDR1 mRNA levels.

The effects of doxorubicin treatment on MDR1 expression in FL5.12 cells were examined. Treatment with doxorubicin resulted in a 3.8-fold decrease in MDR1 mRNAs as 0.61 copies of MDR1 mRNA per million 18S rRNA molecules were observed. In general, treatment with the MEK, rapamycin or nutlin-3a inhibitors did not result in a dramatic decrease in MDR1 levels as between 0.52 to 0.63 MDR1 mRNAs per million 18S rRNA molecule were detected. In contrast, when FL5.12 cells were treated with doxorubicin and the BCL2 inhibitor or the combination of MEK and BCL2 inhibitors, more MDR1 mRNA transcripts were seen (0.91, 1.15 copies respectively) than when the FL5.12 cells treated with doxorubicin by itself. Thus, treatment with the BCL2 inhibitor in the presence of doxorubicin resulted in an approximate to 1.8- to 2.3-fold increase in MDR1 mRNA levels.

Doxorubicin-resistant FL/Doxo cells expressed 3.5-fold higher levels of MDR1, (7.17 copies per million 18S rRNAs), than FL5.12 cells (1.91 copies of MDR1 per million 18S rRNA molecules) (Figures 9 and 10). The levels of these MDR1 transcripts did not appear to be affected by treatment with MEK, BCL2, MEK + BCL2, rapamycin or nutlin-3a treatment when FL/Doxo cells were cultured in the presence of IL-3 and no doxorubicin (5.98 to 6.63 copies of MDR1 per million 18S rRNAs).

The levels of MDR1 transcripts decreased 1.8-fold when FL/Doxo cells were cultured in the presence of IL-3 and doxorubicin (4.28 copies) compared to when the cells were cultured in IL-3 (7.17 copies). When the FL/Doxo cells were treated with the MEK, BCL2, rapamycin and nutlin-3a inhibitors, there were less than 2-fold increase in MDR1 mRNA transcript levels (1.1- to 1.5- fold). The levels of MDR1 increased approximately 2-fold when FL/Doxo cells were cultured in the presence of MEK + BCL2 inhibitors. Thus, the combination of MEK + BCL2 inhibitors and doxorubicin treatment resulted in more MDR1 mRNA transcripts in both FL5.12 and FL/Doxo cells.

The expression of MDR1 was also examined in FL/Doxo + TP53 (DN) cells (Figure 12). When FL/Doxo + TP53 cells were cultured in the presence of IL-3 and no inhibitors, approximately 20 copies of MDR1 were observed per million 18S rRNA molecules. The levels of MDR1 decreased to approximately 15 copies per million 18S rRNA molecules when the cells were treated with MEK + BCL2 inhibitors, rapamycin or nutlin-3A inhibitors.

When FL/Doxo + TP53 (DN) cells were cultured in the presence of IL-3 + doxorubicin and no inhibitors, approximately 13 copies of MDR1 were observed per million 18S rRNA molecules. The levels of MDR1 remained around 12–20 copies per million 18S rRNA molecules when the cells were treated with the inhibitors.

Higher levels of MDR1 transcripts were observed in the FL/Doxo + MEK1(CA) cells. Approximately 40 copies of MDR1 were detected in the FL/Doxo + MEK1(CA) cells when they were cultured with IL-3 and the various inhibitors (Figure 11). Approximately 50 copies of MDR1 per million 18S rRNA molecule were detected when the cells were cultured in the presence of IL-3 and doxorubicin. FL/Doxo + MEK1(CA) cells are the most resistant cells to doxorubicin [21]. The highest levels of MDR1 RNA transcripts were seen in these cells.

### Higher levels of MCL1 expression in doxorubicin-sensitive FL5.12 cells

The levels of the anti-apoptotic MCL1 mRNA transcripts were determined when FL5.12 cells were cultured in the presence and absence of doxorubicin and the signal transduction inhibitors (Figure 9). Approximately 19 copies of MCL1 mRNA transcripts per million 18S rRNA transcripts were detected with FL5.12 cells were cultured in the presence of IL-3. The MCL1 mRNA levels decreased to approximately 7 copies when FL5.12 cells were cultured in the presence of IL-3 and the combination of MEK + BCL2 inhibitor or the nutlin-3a inhibitor. 11 copies of MCL1 mRNA were seen when they were cultured with rapamycin.

Approximately 13 copies of MCL1 mRNA were detected when FL5.12 cells were cultured in the presence of IL-3 + doxorubicin. Treatment of the cells with the MEK, MEK + BCL2, rapamycin or nutlin-3a inhibitors further reduced the levels of MCL1 detected to approximately 6–7 copies per million 18S rRNA molecules.

Approximately 16 copies of MCL1 mRNA per million 18S rRNA molecules were observed in FL/Doxo cells when they were cultured with IL-3 (Figure 10). Treatment of the cells with the MEK, MEK + BCL2, rapamycin or nutlin-3a inhibitors reduced the levels of MCL1 to approximately 8 copies per million 18S rRNA molecules.

When FL/Doxo cells were cultured in the presence of IL-3 + doxorubicin, approximately 14 copies of MCL-1 were detected. Upon treatment of the cells with doxorubicin and the MEK, BCL2, MEK + BCL2, rapamycin or nutlin-3a inhibitors approximately 8–10 copies of BCL2 were observed per million 18S rRNA molecule.

Interestingly lower levels of MCL1 mRNA transcripts were detected in FL/Doxo + TP53 (DN) and FL/Doxo + MEK1 (CA) cells (Figures 11 and 12). Approximately 10 copies of MCL1 mRNA per million 18S rRNA transcripts were observed in FL/Doxo + TP53 (DN) when they were cultured in the presence of IL-3. The expression of MCL1 did not vary significantly when the cells were treated with MEK, BCL2 or rapamycin inhibitors. There was a slight drop to approximately 6 transcripts when the cells were cultured in the presence of MEK + BCL2 inhibitors and to 5.5 transcripts when they were cultured with nutlin-3a. When FL/Doxo + TP53 (DN) were cultured with IL-3 and doxorubicin, approximately 9 copies of MCL1 were detected when the cells were cultured either in the absence or presence of BCL2, rapamycin or nutlin-3a inhibitors. Treatment with the MEK or MEK + BCL2 inhibitors reduced the levels to approximately 6–7 copies of MCL1 per million 18S rRNA molecules.

Approximately 10 copies of MCL1 mRNA per million 18S rRNA transcripts were detected in FL/Doxo + MEK1 (Act) when they were cultured in the presence of IL-3 (Figure 12). The expression of MCL1 varied less than 2-fold when the cells were treated with the MEK, BCL2 or rapamycin inhibitors. There was a slight drop to approximately 6 transcripts when the cells were cultured in the presence of MEK + BCL2 or nutlin-3a inhibitors. When FL/Doxo + MEK1 (Act) were cultured with IL-3 and doxorubicin, approximately 7–11 copies of MCL1 were observed even when the cells were cultured either in the absence or presence of MEK, BCL2, rapamycin or nutlin-3a inhibitors. Treatment with the MEK + BCL2 inhibitors reduced the levels to approximately 6 copies of MCL1 per million 18S rRNA molecules. Thus, the FL5.12 and FL/Doxo cells displayed higher levels of MCL1 mRNA transcripts than the FL/Doxo + TP53 (DN) and FL/Doxo + MEK1(CA) cells.

### Higher levels of BCL2 expression in doxorubicin-sensitive FL5.12 cells

The levels of the anti-apoptotic BCL2 mRNA transcripts were determined when FL5.12 cells were cultured in the presence and absence of doxorubicin and the signal transduction inhibitors (Figure 9). Approximately 20 copies of BCL2 mRNA transcripts per million 18S rRNA transcripts were detected with FL5.12 cells were cultured in the presence of IL-3. The BCL2 levels decreased to approximately 11 copies when FL5.12 cells were cultured in the presence of the MEK inhibitor and 6–7 copies in the combination of MEK + BCL2 inhibitors, rapamycin or the nutlin-3A inhibitors and 11 copies when they were cultured with rapamycin.

When FL5.12 cells were cultured in the presence of IL-3 and doxorubicin, approximately 11 copies of BCL2 were observed. When the cells were cultured in the presence of MEK, MEK + BCL2, rapamycin or nutlin-3a inhibitors approximately 5–8 copies of BCL2 were detected per million 18S rRNA molecules.

Approximately 7 copies of BCL2 per million 18S rRNA molecules were seen in FL/Doxo cells when they were cultured with IL-3 (Figure 10). Treatment of the cells with the MEK, BCL2, MEK + BCL2, rapamycin or nutlin-3a inhibitors reduced the levels of BCL2 to approximately 4 copies per million 18S rRNA molecules.

When FL/Doxo cells were cultured in the presence of IL-3 and doxorubicin, approximately 7 copies of BCL2 were detected. When the cells were cultured in the presence of MEK, BCL-2, MEK + BCL2, rapamycin or nutlin-3a inhibitors approximately 4–5 copies of BCL-2 were observed per million 18S rRNA molecules.

Approximately 10 copies of BCL2 mRNA transcripts per million 18S rRNA transcripts were detected in FL/Doxo + TP53 (DN) cells were cultured in the presence of IL-3 (Figure 11). The BCL-2 levels decreased to approximately 6 copies when FL/Doxo + TP53 (DN) cells were cultured in the presence of the MEK inhibitor and 4 copies in the combination of MEK + BCL2 inhibitors or rapamycin or the nutlin-3a inhibitor.

When FL/Doxo + TP53 (DN) cells were cultured in the presence of IL-3 and doxorubicin, approximately 8 copies of BCL2 were detected. When the cells were cultured in the presence of MEK, MEK + BCL2, rapamycin or nutlin-3a inhibitors, approximately 4–6 copies of BCL2 were observed per million 18S rRNA molecules.

Approximately 5 copies of BCL2 per million 18S rRNA molecules were detected in FL/Doxo + MEK1 (CA) cells when they were cultured with IL-3 (Figure 12). Treatment of the cells with the MEK, BCL2, MEK + BCL2, rapamycin or nutlin-3A inhibitors reduced the levels of BCL2 to approximately 2–3 copies per million 18S rRNA molecules.

When FL/Doxo + MEK1(CA) cells were cultured in the presence of IL-3 and doxorubicin, approximately 5 copies of BCL2 were detected. When the cells were cultured in the presence of MEK, MEK + BCL2, rapamycin approximately 2–3 copies of BCL2 were observed per million 18S rRNA molecules.

### Higher levels of BIM expression in doxorubicin-sensitive FL5.12 cells

The levels of the pro-apoptotic BIM mRNA transcripts were determined when FL5.12 cells were cultured in the presence and absence of doxorubicin and the signal transduction inhibitors (Figure 9). Approximately 25 copies of BIM mRNA transcripts per million 18S rRNA transcripts were detected when FL5.12 cells were cultured in the presence of IL-3. The BIM levels increased to approximately 42 copies when FL5.12 cells were cultured in the presence of the MEK inhibitor and 32 copies in the BCL2 inhibitor treated cells. When the FL5.12 cells were cultured in combination of MEK + BCL2 inhibitors, rapamycin or the nutlin-3A inhibitor, approximately 20 copies of BIM mRNA per million 18S rRNA molecules were observed.

When FL/Doxo cells were cultured in the presence of IL-3 and doxorubicin, approximately 8 copies of BIM were detected (Figure 10). When the cells were cultured in the presence of MEK, BCL2, rapamycin or nutlin-3a approximately 8–9 copies of BIM were detected per million 18S rRNA molecules. When FL/Doxo cells were cultured with the MEK + BCL2 combined inhibitor treatment, approximately 11 copies of BIM were seen.

Approximately 10 copies of BIM per million 18S rRNA molecules were detected in FLDoxo cells when they were cultured with IL-3. Treatment of the cells with the MEK, BCL2, MEK + BCL2, rapamycin or nutlin-3A inhibitors did not significantly alter the levels of BIM mRNA transcripts seen.

Approximately 10 copies of BIM mRNA transcripts per million 18S rRNA transcripts were detected in FL/Doxo + TP53 (DN) cells when they were cultured in the presence of IL-3. The BIM levels remained relatively constant when FL/Doxo + TP53 (DN) cells were cultured in the presence of the MEK, rapamycin or nutlin-3A inhibitors (7–9 copies).

When FL/Doxo + TP53 (DN) cells were cultured in the presence of IL-3 and doxorubicin, approximately 11 copies of BCL2 were observed (Figure 11). When the cells were cultured in the presence of MEK, MEK + BCL2, rapamycin or nutlin-3a inhibitors approximately 10 copies of BIM were detected per million 18S rRNA molecules. The levels of BIM seen in the BCL2 inhibitor-treated FL/Doxo + TP53 (DN) cells cultured in doxorubicin increased slightly to approximately 14 copies per million 18S rRNA molecules.

Approximately 5 copies of BIM per million 18S rRNA molecules were detected in FL/Doxo + MEK1 (CA) cells when they were cultured with IL-3 (Figure 12). Treatment of the cells with the MEK, BCL2, MEK + BCL2, rapamycin or nutlin-3A inhibitors did not alter the levels of BIM detected.

When FL/Doxo + MEK1(CA) cells were cultured in the presence of IL-3 and doxorubicin, approximately 6 copies of BIM were observed. When the cells were cultured in the presence of MEK, BCL2, MEK + BCL2 inhibitors, approximately 9–10 copies of BCL2 were seen per million 18S rRNA molecules. In contrast, rapamycin treatment did not alter the levels of BIM detected compared to the cells treated with no inhibitor.

### Relatively constant levels of Foxo3A detected after doxorubicin or inhibitor treatment

The levels of the Foxo3a mRNA transcripts were determined when the cells were cultured in the presence and absence of doxorubicin and the signal transduction inhibitors. Approximately 62 copies of Foxo3a mRNA transcripts per million 18S rRNA transcripts were detected when FL5.12 cells were cultured in the presence of IL-3 (Figure 9). The Foxo3a levels increased to approximately 75 copies when FL5.12 cells were cultured in the presence of the MEK inhibitor and 69 copies in the presence of the BCL2 inhibitor. When the FL5.12 cells were cultured in combination of MEK + BCL2 inhibitors or rapamycin, 48–51 copies of Foxo-3a were detected. When the cells were cultured with the nutlin-3A inhibitor approximately 64 copies of Foxo-3a per million 18S rRNA molecules were observed.

When FL5.12 cells were cultured in the presence of IL-3 and doxorubicin, approximately 40 copies of Foxo-3a were detected. When the cells were cultured in the presence of MEK, BCL2, or rapamycin inhibitors approximately 41–48 copies of Foxo-3a were detected per million 18S rRNA molecules. The nutlin-3a inhibitor treatment resulted in an increase of Foxo-3a transcripts to 65 copies per million rRNA molecules.

Approximately 48 copies of Foxo-3a per million 18S rRNA molecules were detected in FL/Doxo cells when they were cultured with IL-3 (Figure 10). Treatment of the cells with the MEK, BCL2, MEK + BCL2, rapamycin or nutlin-3A inhibitors resulted in a range of Foxo-3a mRNA transcripts from 34 transcripts with the BCL2 inhibitor to 60 transcripts with the nutlin-3a inhibitor.

When FL/Doxo cells were cultured in the presence of IL-3 and doxorubicin, approximately 35 copies of Foxo-3a were detected. When the cells were cultured in the presence of MEK or BCL2 inhibitors, approximately 37–40 transcripts were detected. 60 Foxo-3a transcripts were observed upon the combined MEK and BCL2 inhibitor treatment, 56 after rapamycin and 53 after nutlin-3a treatment.

Approximately 39 copies of Foxo-3a mRNA transcripts per million 18S rRNA transcripts were detected in FL/Doxo + TP53 (DN) cells were cultured in the presence of IL-3 (Figure 11). The levels of Foxo-3a mRNA transcripts increased to 52, 56, 45, 47 and 49 after MEK, BCL2, MEK + BCL2, rapamycin and nutlin-3a treatment respectively.

When FL/Doxo + TP53 (DN) cells were cultured in the presence of IL-3 and doxorubicin, approximately 40 copies of Foxo-3a were detected. When the cells were cultured in the presence of MEK, BCL2, MEK + BCL2, rapamycin or nutlin-3a approximately 45, 55, 45, 52 and 47 copies of Foxo-3a were observed per million 18S rRNA molecules.

Approximately 27 copies of Foxo-3a per million 18S rRNA molecules were detected in FL/Doxo + MEK1 (CA) cells when they were cultured with IL-3 (Figure 12). Treatment of the cells with the MEK, BCL2, MEK + BCL2, rapamycin or nutlin3A inhibitors did not alter the levels of Foxo-3a as 28, 32, 32 and 37 transcripts were observed respectively.

When FL/Doxo + MEK1(CA) cells were cultured in the presence of IL-3 and doxorubicin, approximately 51 copies of Foxo-3a were detected. When the cells were cultured in the presence of MEK, BCL2, MEK + BCL2, rapamycin and nutlin-3a inhibitors approximately 50, 73, 70, 54 and 64 copies of Foxo-3a were detected per million 18S rRNA molecules.

### BAX mRNA levels are decreased in FL/Doxo + TP53 (DN) and FL/Doxo+MEK1(CA) cells

The levels of BAX mRNA transcripts were determined when the cells were cultured in the presence and absence of doxorubicin and the signal transduction inhibitors. Approximately 76 copies of BAX mRNA transcripts per million 18S rRNA transcripts were detected when FL5.12 cells were cultured in the presence of IL-3 (Figure 9). The BAX levels decreased to approximately 69 copies when FL5.12 cells were cultured in the presence of the MEK inhibitor and 62 copies in the BCL2 inhibitor. When the FL5.12 cells were cultured in combination of MEK + BCL2, rapamycin or nutlin-3A inhibitors, approximately 48 copies of BAX were observed.

When FL5.12 cells were cultured in the presence of IL-3 and doxorubicin, approximately 52 copies of BAX were detected. When the cells were cultured in the presence of the MEK inhibitor, 35 copies were observed. 66 copies of BAX mRNA transcripts were seen when the cells were cultured in the presence of the BCL2 inhibitor. 57 copies of BAX mRNA transcripts were seen when cells were cultured with both MEK and BCL2 inhibitors, 54 and 52 copies were detected when the cells were cultured with rapamycin or nutlin-3A inhibitors, respectively.

Approximately 86 copies of BAX per million 18S rRNA molecules were detected in FL/Doxo cells when they were cultured with IL-3 (Figure 10). Treatment of the cells with the MEK, BCL2, MEK + BCL2, rapamycin or nutlin-3A inhibitors resulted in a range of BAX mRNA transcripts from 46–56 with 46 BAX transcripts observed with the MEK + BCL2 inhibitor to 56 transcripts when the cells were treated with rapamycin.

When FL/Doxo cells were cultured in the presence of IL-3 and doxorubicin, approximately 63 copies of BAX were detected. When the cells were cultured in the presence of MEK or BCL2 inhibitors, approximately 63 transcripts were observed. 57 BAX transcripts were seen upon the combined MEK + BCL2 inhibitor or rapamycin treatment and 60 BAX mRNA transcripts after nutlin-3a treatment.

The TP53 (DN) gene suppressed BAX expression. Approximately 22 copies of BAX mRNA transcripts per million 18S rRNA transcripts were detected when FL/Doxo + TP53 (DN) cells were cultured in the presence of IL-3 (Figure 11). The levels of BAX mRNA transcripts increased to 22, 24, 16, 23 and 26 after MEK, BCL2, MEK + BCL2, rapamycin and nutlin-3a treatment respectively.

When FL/Doxo + TP53 (DN) cells were cultured in the presence of IL-3 and doxorubicin, approximately 24 copies of BAX were observed (Figure 12). When the cells were cultured in the presence of MEK, BCL2, MEK + BCL2, rapamycin or nutlin-3a approximately 21, 19, 17, 25 and 26 copies of BAX were detected per million 18S rRNA molecules.

Approximately 30 copies of BAX per million 18S rRNA molecules were detected in FL/Doxo + MEK1 (CA) cells when they were cultured with IL-3 (Figure 12). Treatment of the cells with the MEK, BCL2, rapamycin or nutlin-3A inhibitors did not alter the levels of BAX mRNA transcripts as 30–32 transcripts were observed. 23 BAX mRNA transcripts were observed in the combined treatment with MEK + BCL2 inhibitors.

When FL/Doxo + MEK1(CA) cells were cultured in the presence of IL-3 and doxorubicin, approximately 30 copies of BAX were detected. When the cells were cultured in the presence of MEK, BCL2, MEK + BCL2 and rapamycin inhibitors, approximately 28, 28, 24 and 32 copies of BAX mRNA transcripts were seen respectively per million 18S rRNA molecules. When the cells were cultured in IL-3 and doxorubicin and nutlin-3, 38 copies of BAX mRNA transcripts were detected.

In summary, the expression of BAX mRNAs was sensitive to inhibitor treatments in FL5.12 and FL/Doxo cells when they were cultured in IL-3. The detection of BAX mRNA transcripts were lower in the FL/Doxo + TP53(DN) and FL/Doxo + MEK1(CA) cells. The nutlin-3A treatment resulted in increased BAX mRNA levels as 38 copies of BAX were detected when the FL/Doxo + MEK1(CA) cells were cultured in the presence of IL-3, doxorubicin and nutlin-3a, while only 30 copies of BAX were detected when the FL/Doxo + MEK1(CA) cells were cultured in the presence of IL-3 and nutlin-3a, BAX mRNA transcripts were inhibited by TP53 (DN) in FL/Doxo + TP53 (DN) cells.

### YY1 mRNA levels are elevated in FL5.12 cells

YY1 is an important transcription factor implicated in many cancers and drug resistance [83, 84]. The expression of YY1 is regulated by TP53 and miR-34 [85–87]. The levels of YY1 mRNA transcripts were determined when the cells were cultured in the presence and absence of doxorubicin and the signal transduction inhibitors. Approximately 94 copies of YY1 mRNA transcripts per million 18S rRNA transcripts were detected with FL5.12 cells were cultured in the presence of IL-3 (Figure 9). The YY1 mRNA levels decreased slightly to approximately 77 copies when FL5.12 cells were cultured in the presence of the MEK inhibitor and 62 copies in the presence of the BCL2 inhibitor. When the FL5.12 cells were cultured in combination of MEK + BCL2 inhibitors, rapamycin or nutlin-3A, approximately 54, 47 and 44 copies of YY1 mRNA transcripts were observed. Thus, the expression of YY1 mRNA was suppressed when FL5.12 cells were cultured with certain inhibitors in the presence of IL-3.

When FL5.12 cells were cultured in the presence of IL-3 and doxorubicin, approximately 52 copies of YY1 mRNA transcripts were detected. When FL5.12 cells were cultured in the presence of the MEK inhibitor, 37 copies were observed. 54 copies of YY1 were detected when the cells were cultured in the presence of the BCL2 inhibitor. 54 copies of YY1 were seen when FL5.12 cells were cultured with both MEK and BCL2 inhibitors, 28 and 25 copies were detected when the FL5.12 cells were cultured with rapamycin or nutlin-3A, respectively.

Approximately 43 copies of YY1 per million 18S rRNA molecules were detected in FL/Doxo cells when they were cultured with IL-3 (Figure 10). Treatment of the cells with the MEK, BCL2, MEK + BCL2, rapamycin or nutlin-3A inhibitors resulted in a range of YY1 mRNA transcripts from 27 to 38 transcripts with 38, 29, 27, 35 and 27 transcripts respectively.

When FL/Doxo cells were cultured in the presence of IL-3 and doxorubicin, approximately 32 copies of YY1 were detected. When the cells were cultured in the presence of MEK inhibitor, 35 copies were observed and 42 copies were seen in the presence of the BCL2 inhibitor. Approximately 36, 36 and 31 YY1 mRNA transcripts were detected upon the combined MEK + BCL2 inhibitors, rapamycin and nutlin-3A inhibitor treatments respectively.

Approximately 21 copies of YY1 mRNA transcripts per million 18S rRNA transcripts were detected in FL/Doxo + TP53 (DN) cells when they were cultured in the presence of IL-3 (Figure 11). The levels of YY mRNA transcripts were: 21, 24, 16, 14 and 16 after MEK, BCL2, MEK + BCL2, rapamycin and nutlin-3a inhibitor treatments, respectively.

When FL/Doxo + TP53 (DN) cells were cultured in the presence of IL-3 and doxorubicin, approximately 21 copies of YY1 were detected (Figure 11). When the cells were cultured in the presence of MEK, BCL2, MEK + BCL2, rapamycin or nutlin-3a inhibitors, approximately 19, 17, 18, 21 and 20 copies of YY1 were seen per million 18S rRNA molecules respectively.

Approximately 16 copies of YY1 per million 18S rRNA molecules were detected in FL/Doxo + MEK1 (CA) cells when they were cultured with IL-3 (Figure 12). Treatment of the cells with the MEK, BCL2, MEK + BCL2, rapamycin or nutlin-3A inhibitors altered the levels of YY1 slightly as 14–20 transcripts were observed (17, 20, 14, 16 and 14 respectively).

When FL/Doxo + MEK1(CA) cells were cultured in the presence of IL-3 and doxorubicin, approximately 18 copies of YY1 were detected. When the cells were cultured in the presence of MEK, BCL2, MEK + BCL2, rapamycin and nutlin-3A inhibitors approximately 18, 17, 15, 18, and 17 copies of YY1 were observed respectively per million 18S rRNA molecules. The expression of YY1 mRNA was higher in FL5.12 cells than in doxorubicin-resistant cells. The more drug resistant cells, FL/Doxo + TP53 (DN) and FL/Doxo + MEK1(CA) expressed less YY1 transcripts than FL/Doxo cells which in turn expressed less YY1 transcripts than FL5.12 cells.

### BCLXL mRNA levels are elevated in FL5.12 cells

The levels of BCLXL mRNA transcripts were determined when FL5.12 cells were cultured in the presence and absence of doxorubicin and the signal transduction inhibitors (Figure 9). Approximately 145 copies of BCLXL mRNA transcripts per million 18S rRNA transcripts were detected with FL5.12 cells were cultured in the presence of IL-3 and 150 when the cells were cultured in the presence of IL-3 and the BCL2 inhibitor. The BCLXL levels decreased to approximately 72 copies when FL5.12 cells were cultured in the presence of the MEK inhibitor. When the FL5.12 cells were cultured in combination of MEK + BCL2 inhibitors, rapamycin or nutlin-3a, approximately 48, 64 and 61 copies of BCLXL mRNAs per million 18S rRNA transcripts were observed. Thus, the expression of BCLXL mRNAs was suppressed when FL5.12 cells were cultured with certain inhibitors in the presence of IL-3. The MEK inhibitor had the greatest suppressive effect on BCLXL mRNAs.

When FL5.12 cells were cultured in the presence of IL-3 and doxorubicin, approximately 88 copies of BCLXL were detected (Figure 9). When the cells were cultured in the presence of the MEK inhibitor, 51 copies were seen. 102 copies of BCLXL mRNA transcripts were seen when the cells were cultured in the presence of the BCL2 inhibitor. 50 copies of BCLXL mRNA transcripts were detected when cells were cultured with both MEK and BCL2 inhibitors, 44 and 54 copies of BCLXL mRNA transcripts were observed when the cells were cultured with rapamycin or nutlin-3A respectively.

Approximately 98 copies of BCLXL per million 18S rRNA molecules were detected in FL/Doxo cells when they were cultured with IL-3 (Figure 10). Treatment of the cells with the MEK, BCL2, MEK + BCL2, rapamycin or nutlin-3a inhibitors resulted in a range of BCLXL mRNA transcripts from 54 to 73 transcripts with 62, 73, 64, 54 and 73 respectively. Rapamycin had the largest suppressive effect on BCLXL mRNAs in FL/Doxo cells cultured in the presence of IL-3.

When FL/Doxo cells were cultured in the presence of IL-3 and doxorubicin, approximately 73 copies of BCLXL mRNA transcripts were detected. When the cells were cultured in the presence of the MEK inhibitor, 53 copies were detected and 48 copies were observed in the presence of the BCL2 inhibitor. Approximately, 125, 57 and 56 BCLXL mRNA transcripts were observed upon the combined MEK and BCL2 inhibitor, rapamycin and nutlin-3a treatment, respectively.

Approximately 38 copies of BCLXL mRNA transcripts per million 18S rRNA transcripts were detected in FL/Doxo + TP53 (DN) cells, when they were cultured in the presence of IL-3 (Figure 11). The levels of BCLXL mRNA transcripts were: 32, 53, 23, 28 and 40 after MEK, BCL2, MEK + BCL2, rapamycin and nutlin-3a treatment respectively.

When FL/Doxo + TP53 (DN) cells were cultured in the presence of IL-3 and doxorubicin, approximately 42 copies of BCLXL were detected. When the cells were cultured in the presence of MEK, BCL2, MEK + BCL2, rapamycin or nutlin-3a inhibitors, approximately 30, 48, 29, 32 and 34 copies of BCLXL mRNAs were observed per million 18S rRNA molecules.

Approximately 31 copies of BCLXL mRNA per million 18S rRNA molecules were detected in FL/Doxo + MEK1 (CA) cells when they were cultured with IL-3 (Figure 12). Treatment of the cells with the MEK, BCL2, MEK + BCL2, rapamycin or nutlin-3A inhibitors altered the levels of BCLXL as 19–33 transcripts were observed (19, 33, 22, 21 and 28) respectively.

When FL/Doxo + MEK1 (CA) cells were cultured in the presence of IL-3 and doxorubicin, approximately 61 copies of BCLXL were detected. When the cells were cultured in the presence of MEK, BCL2, MEK + BCL2, rapamycin and nutlin-3a inhibitors approximately 34, 74, 38, 34, and 41 copies of BCLXL mRNA transcripts were observed respectively per million 18S rRNA molecules. Treatment of FL/Doxo + MEK1 (CA) cells with BCL2 inhibitor prevented the decrease in BCLXL mRNA levels observed in the other inhibitor and IL-3 and doxorubicin treated cells.

The expression of BCLXL mRNA was in general higher in FL5.12 cells than in doxorubicin-resistant cells. The MEK1 inhibitor suppressed BCLXL mRNA levels. An exception was with FL/Doxo cells treated with doxorubicin and both the MEK + BCL2 inhibitors.

### MRP1 mRNA levels are elevated in FL5.12 cells

The levels of MRP1 mRNA transcripts were determined when FL5.12 cells were cultured in the presence and absence of doxorubicin and the signal transduction inhibitors (Figure 9). Approximately 211 copies of MRP1 mRNA transcripts per million 18S rRNA transcripts were detected when FL5.12 cells were cultured in the presence of IL-3 and 225 when the cells were cultured in the presence of IL-3 and the BCL2 inhibitor. The MRP1 levels decreased to approximately 186 copies when FL5.12 cells were cultured in the presence of the MEK inhibitor. When the FL5.12 cells were cultured in combination of MEK + BCL2 inhibitors, rapamycin or nutlin-3a approximately, 116, 96 and 93 copies of MRP1 mRNAs per million 18S rRNA transcripts were observed. Thus, the expression of MRP1 mRNA was suppressed when FL5.12 cells were cultured with certain inhibitors in the presence of IL-3.

When FL5.12 cells were cultured in the presence of IL-3 and doxorubicin, approximately 143 copies of MRP1 were detected. When the cells were cultured in the presence of the MEK inhibitor, 106 copies were detected. 156 copies of MRP1 mRNA transcripts were seen when the cells were cultured in the presence of the BCL2 inhibitor. 112 copies of MRP1 mRNA transcripts were observed when cells were cultured with both MEK and BCL2 inhibitors. 98 and 108 copies of MRP1 mRNA transcripts were detected when the cells were cultured with rapamycin or nutlin-3a respectively.

Approximately 249 copies of MRP1 per million 18S rRNA molecules were detected in FL/Doxo cells when they were cultured with IL-3 (Figure 10). Treatment of the cells with the MEK, BCL2, MEK + BCL2, rapamycin or nutlin-3a inhibitors resulted in a range of MRP1 mRNA transcripts from 178 to 212 transcripts with 212, 190, 194, 178 and 192 respectively. Rapamycin had the largest suppressive effect on MRP1 mRNAs in FL/Doxo cells cultured in the presence of IL-3.

When FL/Doxo cells were cultured in the presence of IL-3 and doxorubicin, approximately 181 copies of MRP1 mRNA transcripts were detected. When the cells were cultured in the presence of MEK inhibitor, 197 copies were observed and 185 copies were detected in the presence of the BCL2 inhibitor. Approximately 226, 200 and 196 MRP1 mRNA transcripts were seen upon the combined MEK and BCL2 inhibitor, rapamycin and nutlin-3a treatment.

Approximately 107 copies of MRP1 mRNA transcripts per million 18S rRNA transcripts were detected when FL/Doxo + TP53 (DN) cells were cultured in the presence of IL-3. The levels of MRP1 mRNA transcripts were: 111, 133, 93, 100 and 89 after MEK, BCL2, MEK + BCL2, rapamycin and nutlin-3a inhibitor treatment respectively.

When FL/Doxo + TP53 (DN) cells were cultured in the presence of IL-3 and doxorubicin, approximately 107 copies of MRP1 were detected (Figure 10). When the cells were cultured in the presence of MEK, BCL2, MEK + BCL2, rapamycin or nutlin-3a inhibitor, approximately 108, 125, 90, 114 and 110 copies of MRP1 were observed per million 18S rRNA molecule.

Approximately 118 copies of MRP1 per million 18S rRNA molecules were detected in FL/Doxo + MEK1 (CA) cells when they were cultured with IL-3. Treatment of the cells with the MEK, BCL2, MEK + BCL2, rapamycin or nutlin-3a inhibitors only slightly altered the levels of MRP1 mRNAs as 92–109 transcripts were observed (108, 93, 92, 96 and 109 respectively).

When FL/Doxo + MEK1 (CA) cells were cultured in the presence of IL-3 and doxorubicin, approximately 174 copies of MRP1 were detected (Figure 12). When the cells were cultured in the presence of MEK, BCL2, MEK + BCL2, rapamycin and nutlin-3a inhibitors, approximately 176, 250, 168, 148, and 173 copies of MRP1 mRNA transcripts were observed respectively per million 18S rRNA molecules. The expression of MRP1 mRNA was higher in FL5.12 and FL/Doxo cells than in FL/Doxo + TP53 (DN) and FL/Doxo + MEK1 (CA) cells. Addition of doxorubicin and the BCL2 inhibitor resulted in higher levels of expression of MRP1 mRNAs in FL/Doxo + MEK1 (CA) cells.

### Levels of beta-2 microglobulin detected

As a control, the levels of beta-2 microglobulin were also examined in all cells and treatment conditions. The levels of beta-2 microglobulin were relatively constant within an individual cell line in the different culture (−/+ doxorubicin) and inhibitor treatments.

### Effects of doxorubicin on YY1 protein expression

**Figure 13:**
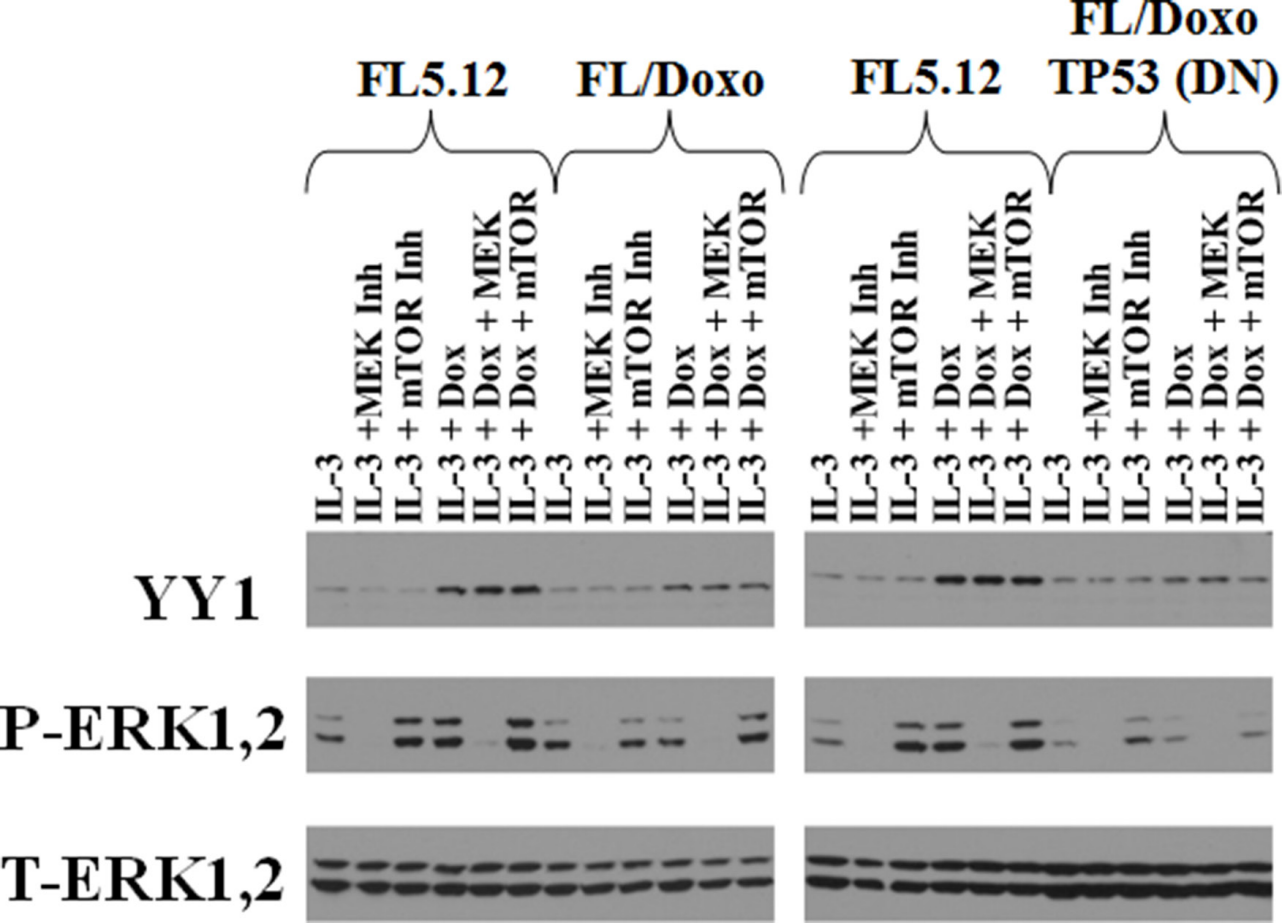
Western blot Analysis of YY1 Expression after IL-3 or IL-3 + Doxorubicin Treatment in FL5.12, FL/Doxo and FL/Doxo + TP53 (DN) Cells Cells were deprived of IL-3 for 24 hours and then treated with nothing, 5,000 nM PD0325901 MEK inhibitor or IL-3 + 100 nM rapamycin mTORC1 blocker for 30 minutes, then the cells were stimulated with either IL-3 or IL-3 + 25 nM doxorubicin for 30 minutes and then cell lysates were prepared and western blot analysis performed as described [21].

The effects of doxorubicin on FL5.12, FL/Doxo and FL/Doxo + TP53 (DN) were determined by western blot analysis (Figure 13). Cells were deprived of IL-3 for 24 hours and then treated with the presence of IL-3 in the presence and absence doxorubicin and MEK or mTORC1 inhibitors. The levels of the expression of activated ERK1,2 were examined as controls. Low levels of YY1 were detected in doxorubicin-sensitive FL5.12 cells, however approximately 10-fold higher levels of YY1 protein were detected when the doxorubicin-sensitive cells were also treated with doxorubicin.

Low levels of YY1 protein were detected in both the FL/Doxo and FL/Doxo + TP53 (DN) cells. The levels of YY1 increased approximately 2-fold in FL/Doxo cells when they were treated with both IL-3 and doxorubicin. In contrast, the levels of YY1 protein did not increase as much in FL/Doxo + TP53 (DN) when they were treated with the combination of IL-3 and doxorubicin, less than 2-fold.

The levels of the YY1 protein did not appear to be sensitive to treatment with either the MEK or mTORC1 inhibitor rapamycin. In contrast, the levels of activated ERK1,2 were sensitive to the MEK inhibitor but did not decrease after treatment with the mTORC1 inhibitor. The levels of activated ERK1,2 actually increased in the rapamycin treated FL5.12, FL/Doxo and FL/Doxo + TP53 (DN) cells. This may have resulted from the feedback loop on ERK1,2 which is elicited by mTORC1 suppression. As a loading control, the levels of total ERK were also monitored in these cells.

## DISCUSSION

**Figure 14:**
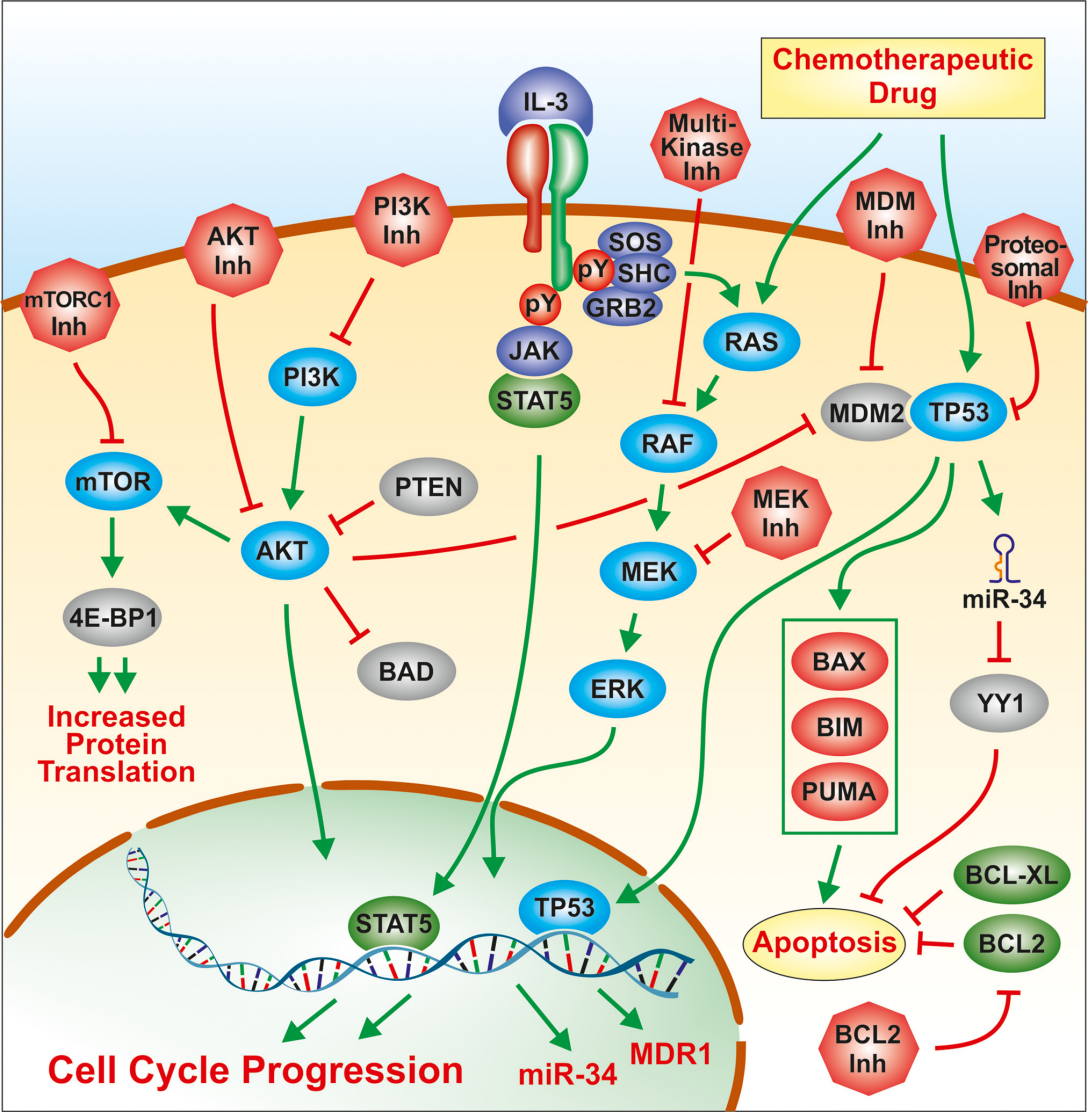
Overview of signal transduction pathways and signaling molecules examined in this study

In our studies, we have investigated the sensitivities of a parental and three doxorubicin-resistant derivative cell lines to various targeted therapeutics, either alone or in combination with doxorubicin. Some of the effects that certain of these targeted therapeutics have on gene expression was also examined in the panel of cells in the presence and absence of doxorubicin. An overview of where some of these signal transduction inhibitors and doxorubicin interact with signaling and TP53 pathways is presented in Figure 14.

TP53 is critical in induction of apoptosis, chemotherapeutic drug resistance, aging as well as the responses to target therapy [87–96]. TP53 can regulate various micro-RNAs (miRs) which are important in drug-resistance in various cancers [87, 97, 98]. The regulation of autophagy by TP53 and other factors is important in chemotherapeutic drug- resistance [99].

The cells which were more drug-resistant, FL/Doxo + TP53 (DN) and FL/Doxo + MEK1(CA) cells were more resistant to the proteasomal inhibitor MG-132 than either the FL5.12 or FL/Doxo + MEK1 (CA) cells (Figure 1A) where are more drug-sensitive than either of the more drug-resistant cells. Inhibition of the proteasome would be predicted to stabilize TP53. This could result in increased TP53 activity and chemosensitization [100].

Proteasomal inhibitors may be combined with certain drugs to increase the effectiveness of acute myeloid leukemia (AML) therapy [101]. Inhibition of the proteasome increased the effectiveness of volasertib and in inducing mitotic arrest and prolonged patient survival [102].

One of the consequences of doxorubicin treatment of certain cancer cell types such as breast cancer and others is the induction of NF-kappaB. Indeed in our studies, we observed that doxorubicin increased the levels of YY1 expression which is an NF-kappa-B-responsive gene [84–87]. Certain second generation proteasomal inhibitors such as carfilzomib will suppress NF-kappaB activity and prevent doxorubicin and proteasomal inhibitor-resistance. One of the molecules induced by doxorubicin and carfilzomib treatment is JNK while one of the events suppressed by the treatment is IkappaBalpha degradation. Carfilzomib treatment overcomes bortezomib-resistance of certain breast cancer cells. Thus, the proteasomal inhibitor carfilzomib may sensitize breast cancer cells to doxorubicin [103].

Interestingly, the doxorubicin-sensitive cell line, FL/Doxo was more sensitive to the MDM2 inhibitor nutlin-3a, than the parent or drug-resistant FL/Doxo + TP53 (DN) and FL/Doxo + MEK1 (CA) cells (Figure 1B). Various additional novels approaches are being used to target MDM-2 [104]. In addition, the targeting of mutant TP53 is being examined, including clinically trials [105].

At the concentrations used, the multi-kinase inhibitor sorafenib did not appear to have significant effects of any of the four cell lines (Figure 2A). The effects of sorafenib on drug resistance of various cancer types have been examined, such as hepatocellular carcinoma (HCC) [106–111].

Not surprisingly, the FL/Doxo + MEK1(CA) cells were the most sensitive to the MEK1 inhibitor (Figure 2B). The FL/Doxo + TP53 (DN) were more sensitive to the MEK1 inhibitor than either FL5.12 or FL/Doxo cells. Thus, the more doxorubicin-resistant cells [FL/Doxo + MEK1 (CA) and FL/Doxo + TP53 (DN) cells were more sensitive to the MEK1 inhibitor than the cells that were not as drug-resistant. In general, the four cell lines were not very sensitive to the JNK inhibitor SP600125 (Figure 2C), although the FL/Doxo + MEK1 (CA) cells were more sensitive than the three other cell lines. This may have resulted between a cross-talk between JNK and MEK signaling in these cells. Interactions between the RAF/MEK/ERK and JNK signaling pathways have been previously reported [88].

JNK signaling is involved in the drug-resistance of many cancers including HCC [112, 113]. The JNK signaling pathway is important in the development and progression of colorectal cancer and other cancer types [114].

The JNK pathway has been investigated in AML and has been determined to be the effector pathway in response to certain drug treatments [115]. The JNK pathway is also critical in pancreatic cancer stem cells (CSC) as suppression of JNK signaling renders the pancreatic CSC sensitive to TNF-related apoptosis-inducing ligand (TRAIL) [116]. JNK regulates Foxo-3a in certain cancer types such as lung adenocarcinoma [117].

The RAF/MEK/ERK pathway is also involved in the drug resistance of various cancers [9, 13, 14, 20, 21, 42]. Various inhibitors, in different combinations, have been shown to influence chemotherapeutic drug-resistance which may occur by altering signaling cross-talk [32]. The RAF/MEK/ERK and other signaling pathways may be targeted in certain cancers in the absence of obvious activated or inactivated oncogenes [14, 15].

The protein phosphatase 2A (PP2A) interacts with the RAF/MEK/ERK and other signaling pathways. Suppression of PP2A activity has been associated with poor patient outcome with certain breast cancer patients as well as doxorubicin resistance [118]. An additional phosphatase, dual-specificity phosphatase 2 (DUSP2) also regulates the RAF/MEK/ERK pathway. Hypoxia inducible factor-1 alpha (HIF1-alpha) has been shown to induce lapatinib-resistance in HER2-postive breast cancers by deregulating DUSP2 activity [119]. Protein phosphatases often function as tumor suppressor proteins [65, 69].

Targeting of the RAF/MEK/ERK, PI3K/PTEN/AKT/mTORC1, SRC or other signaling pathways has shown promise in anti-cancer therapy [42]. Targeting MEK (and downstream ERK) with PD0325901 has been shown to enhance the ability of the PI3K inhibitor NVP-BKM-120 in lung cancer cells. NVP-BKM-120 induces ERK activation and autophagy. Suppression of ERK activation resulted in increased therapeutic effectiveness against the lung cancer cells [120]. The effectiveness of PD0325901 has been examined in cells which proliferate in response to mutant HRAS and was determined to be effective in suppressing growth, especially in combination with mTOR inhibitors [121]. Combining PD0325901 and SRC inhibitors suppressed tumor growth and induced mesenchymal to epithelial transition in non-small cell lung cancer cells [122].

The effects of the PI3K inhibitor were more significant on the more drug-resistant cells as the IC_50_s for LY294002 were higher in FL5.12 and FL/Doxo cells than in FL/Doxo + MEK1 (CA) and FL/Doxo + TP53 (DN) cells (Figure 3A). Thus, the degree of drug-resistance was correlated with sensitivity to the PI3K inhibitor, LY294002. The concept of targeting MEK and PI3K is being examined in many cancers including those that are drug-resistant [42, 123].

The PI3K/PTEN/AKT/mTORC1 pathway is dysregulated in multiple cancers including leukemia and multiple solid cancers. Often components of this and related phospholipid signaling pathways are tumor suppressors [124, 125]. Multiple components of the PI3K/PTEN/AKT/mTORC1 pathway have and are being considered for potential targeting in human cancer, e.g., p70S6K in breast cancer [126], as well as, obesity [127].

Certain PI3K inhibitors are being investigated in drug-resistance hematopoietic and other cancer types and are showing promising effects [53, 54, 128–131]. Combining PI3K/mTOR and other pathway inhibitors or glucocorticoids may be an approach to overcome the therapeutic-resistance of certain cancers [132–134]. Targeting the PI3K and androgen receptor pathways may be an approach to treat therapy-resistant prostate cancer [135, 136].

The doxorubicin-sensitive FL5.12 cells were more sensitive to the AKT inhibitor A443654 than the doxorubicin-resistant FL/Doxo, FL/Doxo + MEK1 (CA) and FL/Doxo + TP53 (DN) cells (Figure 3B). AKT has been examined as a therapeutic target in various leukemia and other cell types [43, 48, 51, 137].

Strikingly, the doxorubicin-resistant FL/Doxo cells were more sensitive to the mTORC1 blocker rapamycin than the FL5.12, FL/Doxo + MEK1 (CA) and FL/Doxo + TP53 (DN) cells (Figure 3C). The FL/Doxo cells displayed a very low IC_50_ to rapamycin. The FL/Doxo + TP53 (DN) were relatively resistant to the mTORC1 blocker. Interactions between suppression of TP53 and sensitivity to rapamycin have been previously reported [138, 139]. One of the side effects of rapamycin treatment is activation of ERK [140]. Suppressing mTORC may inhibit aspects of cellular senescence [141, 142]. Combining mTOR inhibitors with other drugs has shown promise in treatment of certain drug-resistant cancers [45, 46, 143]. Resistance to rapamycin in certain solid cancers has been associated with epithelial to mesenchymal transition (EMT) [144].

The FL/Doxo cells were also very sensitive to the ABT-737 BCL2 inhibitor, while the FL/Doxo + MEK1(CA) were more resistant (Figure 3D). Thus, cells which express activated MEK1 were resistant to the BCL2 inhibitor. Novel BCL2 antagonists are being developed to suppress leukemia as well as drug resistance of other cancers [24, 145–148]

The effects of combinations of signal transduction inhibitors in suboptimal concentrations to reduce the IC_50_ for doxorubicin were examined (Figure 4). The combination of doxorubicin and MG132 reduced the doxorubicin IC_50_ in FL5.12 and FL/Doxo cells dramatically. In contrast, the combination of doxorubicin and MG-132 reduced the doxorubicin IC_50_ less in the more doxorubicin-resistant FL/Doxo + TP53 (DN) and FL/Doxo + MEK1 (CA) (Figure 4A).

Addition of a suboptimal concentration of the MEK inhibitor PD0325891 did not have dramatic effects on the doxorubicin IC_50_ (Figure 4B). In contrast, introduction of a suboptimal concentration of rapamycin did reduce the IC_50_ of all cell lines, especially in the FL5.12, FL/Doxo and FL/Doxo + MEK1 (CA) cells. These results document the importance of the mTORC1 pathway in the response to doxorubicin.

The combination of doxorubicin and suboptimal concentrations of the MDM2 inhibitor nutlin-3a yielded interesting results as nutlin-3 sensitized cells with wild type (WT) TP53 (FL5.12, FL/Doxo and FL/Doxo + MEK1 (CA) but not so much in FL/Doxo + TP53 (DN) cells (Figure 4D). The FL/Doxo + TP53 (DN) cells still have some endogenous WT TP53.

Interestingly, the BCL2 inhibitor ABT-737 sensitized all the cell Ines to doxorubicin (Figure 5A). Likewise, addition of a suboptimal dose of doxorubicin sensitized all the cell lines to ABT-737. These results indicate the importance of BCL2 and other BCL2-related proteins (*e.g*., BCLXL) that are inhibited by the ABT-737 inhibitor in the response to rapamycin.

Addition of suboptimal concentrations of the BCL2 inhibitor did sensitize the doxorubicin resistant FL/Doxo, FL/Doxo + TP53 (DN) and FL/Doxo + MEK1 (CA) cells to the PI3K inhibitor LY294002 more than FL5.12 cells (Figure 5A). Likewise, addition of suboptimal concentrations of PI3K inhibitor LY294002 did sensitize doxorubicin-resistant FL/Doxo, FL/Doxo + TP53 (DN) and FL/Doxo + MEK1 (CA) cells to the ABT-737 BCL2 inhibitor more than FL5.12 cells (Figure 5B). These results point to the importance of BCL2 in the response to PI3K inhibitors. The PI3K/PTEN/Akt/mTORC pathway is very important in governing the drug-resistance of various cancers [149].

Addition of suboptimal concentrations of the MEK inhibitor did sensitize the doxorubicin-resistant FL/Doxo, FL/Doxo + TP53 (DN) and FL/Doxo + MEK1 (CA) cells to the BCL2 inhibitor ABT-737 more than FL5.12 cells (Figure 6A). Addition of suboptimal concentrations of BCL2 inhibitor ABT-737 did sensitize-doxorubicin-resistant FL/Doxo and parental FL5.12 cells to the MEK inhibitor PD0325901 more than FL5.12 cells. These results point to the importance of BCL2 and related family members in the response to MEK inhibitors.

The proteasomal inhibitor MG132 was able to sensitize FL/Doxo, FL5.12 and FL/Doxo + MEK1 (CA) cells to the effects of rapamycin (Figure 7A). However, addition of a suboptimal dose of the proteasomal inhibitor MG132 did not sensitize the FL/Doxo + TP53 (DN) cells to the mTORC1 inhibitor rapamycin.

Addition of a suboptimal doses (500 nM) of the MDM2 inhibitor (nutlin-3a) did sensitized the: FL/Doxo, FL5.12, FL/Doxo + MEK1 (CA) cells to rapamycin. Less effects were observed with FL/Doxo + TP53 (DN) cells (Figure 7B).

The expression of various apoptotic regulatory genes, MDM2 and activated JNK were examined in the cells in the presence and absence of IL-3 and doxorubicin and the proteasomal inhibitor MG132 by western blot analysis (Figure 8). Previously, we examined the expression of TP53 and activated ERK1,2 in these cells under the same conditions [21]. Low levels of BIM and MDM2 were detected in FL5.12 cells (Figure 8A). In contrast, higher levels of BIM and MDM2 were detected in FL/Doxo cells. Higher levels of MCL1 transcripts were detected in FL5.12 cells upon treatment with 100 and 1000 nM doxorubicin. Interestingly, the levels of MCL1 protein were increased upon treatment of FL5.12 cells and 10 μM MG132. MCL1 was not detected by western blot analysis in FL/Doxo cells. Activated JNK was detected in FL5.12 cells upon treatment with 10 μM MG132. The pro-apoptotic BAX protein was detected in both FL5.12 and FL/Doxo cells. These data demonstrate that the drug-resistant FL/Doxo cells express higher levels of MDM2 and BIM than the drug-sensitive FL5.12 cells.

The expression of various apoptotic regulators, TP53 and activated JNK were examined in FL/Doxo + TP53 (DN) and FL/Doxo + MEK1 (CA) cells (Figure 8). High levels of mutant TP53 were of observed in FL/Doxo + TP53 (DN) cells, while the BAX protein was not detected. In contrast, the TP53 protein was detected upon treatment of FL/Doxo + MEK1 (CA) cells with the proteasomal inhibitor and the BAX protein was detected at high levels. The levels of BIM and activated JNK were low in both FL/Doxo + MEK1 (CA) cells.

The levels of some of these and other gene mRNA transcripts were also measured by qRT-PCR (Figures 9, 10, 11, 12). Higher levels of MDR1 were detected in the doxorubicin-resistant FL/Doxo, FL/Doxo + TP53 (DN) and FL/Doxo + MEK1 (CA) cells than the doxorubicin-sensitive FL5.12 cells. In contrast, higher levels of MCL1, BCLXL, BCL2 and YY1 expression were detected in the doxorubicin-sensitive FL5.12 than the doxorubicin-resistant FL/Doxo, FL/Doxo + TP53 (DN) and FL/Doxo + MEK1 (CA) cells. Relatively similar levels of Foxo-3a mRNA were detected in these studies. In contrast, the presence of TP53 (DN) suppressed the levels of BAX detected in the FL/Doxo + TP53 (DN) cells than the other cell lines. Higher levels of MRP1 were detected in FL5.12 and FL/Doxo cells than in FL/Doxo + TP53 (DN) and FL/Doxo + MEK1 (CA) cells. The results with MRP1 expression were in contrast with the levels of MDR1 expression that indicated more MDR1 expression in the drug-resistant FL/Doxo, FL/Doxo + TP53 (DN) and FL/Doxo + MEK1 (CA) cells. Thus, it is likely that MDR1 plays a bigger role in drug resistance than MRP1 in these cells. Our studies document that one of the genes that is increased in the doxorubicin-resistant cells is MDR1 which encodes an important drug transporter often implicated in drug-resistance [149–152]. MRP1 is also an important protein in drug-resistance [149, 153].

The levels of the YY1 protein increased upon doxorubicin treatment of FL5.12 cells. Doxorubicin may induce TP53 expression which results in increased stability of the YY1 protein. Lower levels of YY1 protein were induced after doxorubicin treatment of FL/Doxo and FL/Doxo + TP53 (DN) cells suggesting that in the doxorubicin-resistant cells less YY1 protein was present after doxorubicin treatment than in the doxorubicin-sensitive cells. In contrast, the levels of total ERK1,2 protein were similar in all cells in the presence and absence of doxorubicin. The stabilization of YY1 protein was determined to be independent of the MEK and mTORC1 pathways as treatment with MEK and mTORC1 inhibitors did not suppress the accumulation of YY1 protein, while treatment with the MEK inhibitor, but not mTORC1 inhibitor did suppress the detection of activated ERK1,2. The NF-kappaB pathway may have effects on the levels of YY1 expression [154].

The effects of the gene status of TP53 and the activity of MEK1 were very important in terms of the sensitivity to certain small molecule inhibitors as well as on gene expression. TP53 normally regulates the expression of numerous miRs that often suppress tumor growth. Thus, inhibition of WT TP53 by introduction of a DN construct often resulted in therapy-resistance. Expression of activated MEK1 also increased drug resistance and in some cases altered the response to certain small molecule inhibitors. Our results point to the complex interactions that can occur by mutations at various oncogenes and tumor suppressor proteins that can alter sensitivity to chemotherapeutic drugs and small molecule signal transduction inhibitors.

## MATERIALS AND METHODS

### Cells and tissue culture conditions

FL5.12, FL/Doxo, FL/Doxo + TP53 (DN) and FL/Doxo + MEK1 (CA) were derived and cultured as described [21]. IL-3 was purified from the IL-3 dependent cell line WEHI-3B as described [21]. MTT proliferation assays were performed as described [10–15, 20, 21, 23].

### Treatment of cells with signal transduction inhibitors and doxorubicin

FL5.12, FL/Doxo, FL/Doxo + TP53 (DN) and FL/Doxo + MEK1 (CA) were titrated with the different signal transduction inhibitors and doxorubicin as described [10–15, 20, 21, 23]. Statistical analysis was performed using GraphPad Prism.

### Western blot analysis

Protein analysis was performed as described with the indicated antibodies [21].

### Quantitative RT-PCR

Total RNA was isolated from FL5.12 and drug resistant derivative cells lines with RNeasy Plus minikits (Qiagen, Valencia, CA) and TRIzol reagent (Invitrogen, Carlsbad, CA) which was reverse transcribed by Superscript III (Invitrogen, Carlsbad, CA). Primers for murine genes were obtained from Applied Biosystems, Foster City, CA. Real-time RT-PCR was performed with TaqMan Gene Expression assays. TaqMan primers are listed in Table 1.

**Table 1:** TaqMan primers used in this study

Official symbol	a.k.a.	TaqMan assay ID
Bcl2l1	Bcl xl	Mm00437783_m1
Mcl1	Mcl1	Mm01257351_g1
Bcl2	Bcl2	Mm00477631_m1
Bax	Bax	Mm00432050_m1
Yy1	yin-yang 1	Mm00456392_m1
Bcl2l11	Bim	Mm01333921_m1
Foxo3a		Mm01185722_m1
Abcb1b	Mdr1	Mm00440736_m1
Abcc1	Mrp	Mm00456156_m1
18S	18S rRNA	Hs99999901_s1
b2m	beta-2-microglobulin	Mm00437762_m1
